# Polyphenolics profile effects upon the antioxidant and antimicrobial activity of propolis extracts

**DOI:** 10.1038/s41598-021-97130-9

**Published:** 2021-10-11

**Authors:** Mădălina Maria Nichitoi, Ana Maria Josceanu, Raluca Daniela Isopescu, Gabriela Olimpia Isopencu, Elisabeta-Irina Geana, Corina Teodora Ciucure, Vasile Lavric

**Affiliations:** 1 Doctoral School “Applied Chemistry and Materials Science”, University Politehnica of Bucharest, Bucharest, Romania; 2 Department of Analytical Chemistry and Environmental Engineering, University Politehnica of Bucharest, Bucharest, Romania; 3 Department of Chemical and Biochemical Engineering, University Politehnica of Bucharest, Bucharest, Romania; 4 National Research and Development Institute for Cryogenics and Isotopic Technologies – ICSI, Ramnicu Valcea, Romania

**Keywords:** Biological techniques, Chemistry, Engineering

## Abstract

Propolis, a complex bee product, is a source of numerous bioactive principles, beneficial for human health, therefore it is intensively studied. In the present work, extracts of propolis from Bihor Romanian County were studied to identify the relationship between the polyphenolic derivatives profile and their antioxidant and antimicrobial activity. Extracts were obtained using water and 25%, 50%, and 70% ethanolic solutions (w/w), at 2:1, 4:1, and 6:1 liquid: solid ratios (w/w). 21 polyphenolic derivatives were quantified by UHPLC-MS, proving that the extracts composition strongly depends on the solvent. The sum of quantified polyphenolics extracted varied between 1.5 and 91.2 mg/g propolis. The antioxidant capacity was evaluated using the free radicals 2,2’-azino-bis (3-ethylbenzothiazoline-6 sulfonic acid) diammonium salt (ABTS) and 1-diphenyl-2-picryl-hydrazyl (DPPH) scavenging methods. Antimicrobial efficiency was tested against Gram-positive (*B. subtilis*), Gram-negative bacteria (*E. coli*), and fungi (*C. albicans*) by disc-diffusion method. All extracts, even the aqueous ones, demonstrated antibacterial and antifungal activity. Chemometric methods (partial least squares) and a saturation-type model were used to evaluate the contribution of various bioactive principles in building the antioxidant capacity of extracts. Both experimental and modelling results show that 50% ethanolic extracts provide a rich polyphenolics profile and ensure a good antioxidant capacity.

## Introduction

The social and economic value of propolis has been demonstrated by addressing health, nutrition, and product-development issues. Access to its potentially useful bioactive principles is restricted by different other plant debris, present in diverse proportions, depending on the collection time and geographical origin^1–4^. The most representative biologically active compounds in temperate zones propolis are phenolic acids and flavonoids^5,6^.

Due to the large extent of wax and debris present in propolis, ethanol or its aqueous mixtures represented the preferred extraction media over the time, given its selectivity the active principles of interest^7–10^. Chloroform, acetone^2^, methanol^11^, hexane^12^, glycerol with 5% (v/v) ethanol or propylene glycol with 2% (v/v) ethanol^13^, olive oil, β-cyclodextrin, 1.5% (w/v) aqueous solution, or 1:1 combination of these^14^ were also employed in macerations at ambient temperature.

Several research groups have studied the effect of experimental factors upon the extraction yield and extracts properties. Temperature raising in stirred vessels^15–17^ or pressurized liquid extraction systems^18^ led to better recovery yields. The liquid: solid ratio (w/w) was a widely varied experimental parameter, ranging from 2500:1 to 3:1^7,11,19–25^. The process was mainly centred on increasing the global yield, without much attention towards the extracted profiles of phenolic acids and flavonoids.

Process intensification, aiming to increase the active principles concentration and to decrease the process time, has been sought by applying ultrasounds^14,20^, microwave fields^20^, or supercritical CO_2_ extraction^16^. Process time decrease was remarkable, the 24 h cycles for ambient conditions extraction with 70% ethanol dropping between 10 s and 45 min cycles^26^, with intermittent cooling, to avoid excessive heating and polyphenolics degradation.

Despite duration, maceration is still the method of choice for many propolis-selling beekeepers, due to its simplicity in terms of operations and equipment. Intensification techniques like sonication, microwaves, pressurized liquid extraction are not considered by most beekeepers, as they come with high initial investment costs and safety operation requirements. Therefore, mastering the traditional maceration to obtain dedicated usage extracts remains imperative in the artisanal apiculture.

The targeted compounds in antibacterial, antifungal, antioxidant and antitumoral effects are present in small and variable amounts in the natural propolis. Pinocembrin, *p*-coumaric acid, 3-acetylpinobanksin, pinobanksin-3-acetate, and caffeic acid were demonstrated to have anti-fungal activity against *C. albicans*^27^. Propolis antimicrobial activities begin to be documented against different bacteria^28^, yeasts^29^, viruses^30^, and parasites^31^. In vitro, propolis may act directly on microorganisms, and in vivo it may stimulate the immune system, activating the mechanisms involved in the microorganisms killing.

Studies on the propolis antiviral activity showed that it can be used as an adjunct in fighting against respiratory infections caused mainly by coronaviruses^32^. Compounds like caffeic acid and caffeic acid phenethyl ester (CAPE), quercetin, kaempferol, *p*-coumaric acid, galangin, chrysin, with anti-inflammatory and immuno-regulatory effects, make propolis a possible significant component in treating various viral diseases, including Covid-19^33^. Kowacz and Pollack^34^ have shown that propolis could also be used as a spray to physically prevent viruses from entering the body by forming an extensive layer of water exclusion zone.

Most of the propolis antioxidant properties were attributed to galangin and pinocembrin^2^. It had been accepted that phenolic compounds in propolis donate hydrogen ions to free radicals, thus hindering lipids, proteins, and nucleic acids oxidation^35^.

Wagh^36^ reported that an aqueous propolis extract presented higher antioxidant effects than ethanolic extracts, with galangin showing more efficiency than pinocembrin, regardless the extraction solvent. Kubiliene et al^37^ compared composition and biological activities of propolis extracts prepared with non-alcoholic solvent mixtures and found no significant differences in the total content of phenolic compounds compared to ethanolic extracts.

Despite the reduced phenolic acids’ and flavonoids’ aqueous solubility, water should not be overlooked as extractant. Fine-tuning the water–ethanol ratio, contact time, and/or solid: liquid ratio could open way to operational parameters customized for yielding extracts with high content of targeted compounds, displaying high antioxidant capacity, and equally high antimicrobial activity.

Composition reports on propolis are extremely diverse, given the specificity of flora, climate, geographical and hydrological conditions at the site of collection. It is an established fact that propolis from temperate zone, called poplar propolis contains mainly phenolic acid, flavonoids, aromatic acids, and their esters^23^. Variability of debris or access to certain processing practices endorse reporting results as mass of extracted matter per weight of ‘balsam’^22^, ethanolic extract of propolis^38^, or crude propolis^20^.

Romanian propolis, as a poplar type propolis, is reported to contain around 300 mg/g polyphenolic compounds^1^, with a large variability according to the provenance region, harvest time, and beekeeping practices. Caffeic acid, chrysin, CAPE, and pinocembrin are most commonly found^6,10,22,38–40^, accompanied by ferulic and *p*-coumaric acids, galangin, kaempferol and quercetin. Apigenin, rutin, luteolin, narigenin, and pinostrobin are less often quantified^20,38,39^.

Experimentally, there should be a quest for better extraction conditions, using environmentally friendly solvents or solvent mixtures. Within this frame, the present work carries on a large experiment involving solvent composition, solid: liquid ratio, and process duration. The detailed composition of the polyphenolic derivatives in propolis, obtained by UHPLC-MS, gives the chance to evaluate the importance of the operating conditions for the extraction of the main bioactive principles which contribute to the antioxidant and antimicrobial activity. Chemometric methods (Partial Least Square regression) were used to emphasize a relationship between the chemical compounds’ nature and extracts antioxidant/antimicrobial activity. A saturation-type model was used to obtain a general insight of the contribution of the polyphenolics profile to the variation of antioxidant capacity.

## Materials and methods

### Propolis samples

Propolis, produced by *Apis Meliffera Carpatica*, was donated by dr. Roxana Spulber, Institute for Research and Development for Beekeeping, Bucharest, Romania; it originated from an apiary registered with the Romanian Beekeepers’ Association in Bihor County, Romania, being harvested by the beekeepers in March-November 2016 season. It was stored in laboratory at − 20 °C until processing and analysis. Prior to extractions, propolis was grounded to fine powder with a Retsch 200 mill (Haan, Germany).

### Reagents

Trolox (95%), 2ʹ-azino-bis(3-ethylbenzothiazoline-6-sulphonic acid)–ABTS–(98%), K_2_S_2_O_8_ (99%), 2,2-diphenyl-1-picryl-hydrazyl (DPPH), quercetin (95%), ethanol (99,8%) were purchased from Sigma-Aldrich (Steinheim, Germany) and used without further purification.

Analytical standards (apigenin, galangin, kaempferol, isorhamnetin, chrysin, pinocembrin, gallic acid, abscisic acid, *p*‐coumaric acid, syringic acid, caffeic acid, caffeic acid phenyl ester—CAPE, chlorogenic acid, ferulic acid, ellagic acid, vanillic acid, *p*-hydroxybenzoic acid, 3,4‐dihydroxybenzoic acid, *t*‐cinnamic acid, (+)–catechin, and (−)–epicatechin), from Sigma-Aldrich (Steinheim, Germany), were used to prepare individual 500 mg/L methanolic stocks. A 10 mg/L mixed working standard in methanol was obtained by appropriate dilution of individual stocks. Automatic pipettes and class A volumetric glass flasks were used.

Gradient grade methanol for liquid chromatography (99.9%), and formic acid (98–100%) were purchased from Merck (Darmstadt, Germany). Aqueous solutions were prepared with deionized water produced by a Milli-Q Millipore system (Bedford, USA).

All chemicals for antimicrobial activity were microbiologically pure. Nutrient agar (NA) was purchased from Sigma–Aldrich (Steinheim, Germany), and yeast extract peptone dextrose (YPD) from Carl Roth (Karlsruhe, Germany).

### Extraction procedures

Water and ethanolic solutions (25%, 50%, 70% w/w) were used for extraction. The liquid:solid ratios (w/w) were 2:1, 4:1, and 6:1. Phases were contacted at room temperature, 150 rpm for 1, 3, and 5 days, in an ES 80-Grant Instruments orbital shaker (UK). Extracts were separated from waxes with Filtrak No 389, Ø 12.5 cm filter paper, and stored at − 20 °C until analysis.

The extracts coding system is an alpha-numerical combination with the letter representing the solvent (A—water, E—25%, EE—50%, EEE—70% ethanol), the first digit, the liquid:solid ratio (**2**:1, **4**:1 and **6**:1), while the second digit stands for the contact duration (**1** for 24 h, **3** for 72 h, **5** for 120 h).

### Polyphenolic derivatives quantification by UHPLC-MS

The phenolic acids and flavonoids were quantified with an UltiMate 3000 UHPLC System (Thermo Fisher Scientific), coupled with a Q Exactive Focus Hybrid Quadrupole‐Orbitrap mass spectrometer equipped with Heated Electrospray Ionisation (HESI) probe (Thermo Fisher Scientific).

The Kinetex C18 column (Fusion-RP, 100 Å, LC Column 100 × 2.1 mm, particle diameter 1.7 µm) was operated at 30 °C. The injection volume was 10 µL, each injection being repeated three times. Mobile phase A contained formic acid, 0.1% aqueous solution, while solution B was a methanolic 0.1% formic acid solution. The gradient elution program started with 100% A: 0–2 min, from 100% A to 98% A, 2% B at 400 µL/min; 2–5 min, from 98% A, 2% B to 50% A, 50% B at 300 µL/min; 5–17 min from 50% A, 50% B to 2% A, 98% B at a 300 µL/min; 17–18 min, from 2% A, 98% B to 98% A, 2% B at 400 µL/min; 18–20 min to 100% A.

Mass spectra were recorded in the negative ionization mode in the 100–800 m/z range, at 70,000 resolution. Nitrogen was used as collision, sheath, and auxiliary gas at 11–48 arbitrary units flow rates. The spray voltage was 2.5 kV and the capillary temperature 320 °C. Energy of the Collision-induced Dissociation cell was varied in the 30–60 eV range. Data were acquired and analysed with the Thermo Xcalibur software package (Version 4.1).

Calibrations were carried out in the 50–1750 μg/L concentration range, by serial dilution of the 10 mg/L methanolic standard mix. Calibration curves parameters are available in Supplementary Table S1, online*,* together with other measurements quality information.

Propolis extracts were filtered through a 0.45 μm polytetrafluoroethylene membrane and diluted before injection into the UHPLC-MS system. Typical chromatograms recorded are presented in Supplementary Figure S1 online. Individual phenolic acids and flavonoids contents were reported as either μg/mL extract or µg/g propolis.

### Antioxidant capacity

Extracts were tested for antioxidant capacity by scavenging the long-lived free radicals ABTS^+^ and DPPH, Trolox being model compound^8^. ABTS assay was carried out 734 nm, against a reagent blank in water, while DPPH assay was performed at 515 nm^41^.

Calibration curve parameters for ABTS assay were: 5.76 ± 0.07 μg/mL slope, 3.76 ± 0.63 μg/mL intercept, 0.9959 coefficient of determination, and 2.07 μg/mL standard error of response. Bias did not exceed 3.8%, with 95.5–101.7% recovery for the investigated concentration range. Calibration curve parameters for DPPH assay were: (− 7.3 ± 0.5) × 10^–3^ μg/mL slope, 0.258 ± 0.009 μg/mL intercept, 0.9956 coefficient of determination, and 0.008 μg/mL standard error of response. Bias did not exceed 5.4%, with 94.9–103.5% recovery for the investigated concentration range.

Propolis extracts were diluted prior reacting with the free radicals’ solution. The antioxidant capacity was calculated, irrespective of the method at hand, as equivalent concentration of Trolox from the calibration curves, namely Trolox Equivalent Antioxidant Capacity (TEAC), and reported as μg TEAC/mL.

### Antimicrobial activity

#### Test strains

Strains used in the tests included Gram-negative bacteria (G^−^), *Escherichia coli* (K12-MG1655) - *E. coli*, Gram-positive bacteria (G^+^), *Bacillus subtilis spizizenii nakamura* (ATCC 6633) - *B. subtilis*, and yeast, *Candida albicans* (ATCC 10,231) - *C. albicans*. The bacterial strains were cultured on NA at 37 °C for 24 h. Bacterial inocula were prepared in sterile saline solution, 0.85% (w/v) NaCl, with the cell density corresponding to 0.5 McFarland (~ 10^8^ CFU/mL). Yeast was cultured on YDP at 28 °C for 48 h. The yeast inoculum was prepared in a sterile solution, 0.85% (w/v) NaCl, to reach an approximately 10^6^ CFU/mL population, using a hemacytometer.

#### Disc-diffusion method

Propolis extracts antimicrobial activity was determined with a disc-diffusion method (ISO/TS 16,782:2016 (en)). The plates with bacteria were incubated at 37 °C for 24 h, and with yeast at 28 °C for 48 h. The diameter of inhibition zones (IZ, mm) around the discs was measured after incubation. The inhibition zones were calculated as differences between the measured diameters of the clear zones and the disc diameter (standard), with the solvent inhibition zone subtraction. All tests were performed in triplicate. The three concentrations obtained for different liquid: solid ratios were used to determine the minimum inhibitory concentration (MIC, µg/mL) for the extractions performed at different time intervals. The inoculum suspensions of the used strains were distributed into a 96-well microtiter plate, containing two-fold serial dilution of samples. MIC value was established as the lowest extract concentration which inhibited bacterial or fungal growth after incubation at optimal temperature^42^.

### Statistical modelling

Statistical modelling was applied to investigate the correlation between the extracts’ polyphenolics content and the antioxidant capacity. A saturation type model (Eq. 1) was assumed, to find out if there is a limiting polyphenolic acids and flavonoids concentration, *c*_*p*_*,* for which the antioxidant capacity, *Q*_*a*_, attains saturation.

1 $$Q_{a} = \frac{{K_{\max } \cdot c_{p} }}{{K_{c} + c_{p} }}$$

*Q*_*a*_ is the antioxidant capacity (TEAC, μg/mL), while *c*_*p*_ is the polyphenolic derivatives concentration (μg/mL). The model parameters are *K*_*max*_, (μg Trolox /mL), the extract antioxidant potential at theoretically very high *c*_*p*_ values (*c*_*p*_ → ∝), and *K*_*c*_ (μg/mL), the critical concentration (*c*_*p*_ for which *Q*_*a*_ is half *K*_*max*_). The model parameters were identified minimizing the objective function (2):

2 $$F = \sum\limits_{i = 1}^{n} {(Q_{a,\exp } - Q_{{a,{\text{mo}} {\text{del}}}} )^{2} }$$

*n* being the number of samples considered.

The saturation model regression against the experimental data was performed for correlating the concentration of chemical compounds identified in each extraction solvent with the corresponding antioxidant capacity of the extract.

Statistical analysis also correlated individual polyphenolics concentration in the extracts with the corresponding antioxidant capacity. Therefore, the link between the antioxidant capacity and the polyphenolic composition identifies the chemical compounds synergistically involved in the antioxidant capacity.

Data correlation was performed by partial least squares (PLS), a combination of Principal Component Analysis (PCA) and regression^43^. PLS is used when the predictors number is larger than the samples number, as in spectral data correlation^44^. These components included, in decreasing order, the variability between samples in terms of polyphenolics composition and antioxidant capacity. The “variable influence on projection” known as “VIP” was used to identify the bioactive principles significantly influencing the antioxidant capacity variation. A VIP score summarizes the contribution of each variable to the regression model. Variables with VIP values larger than 1 are generally considered relevant. The VIP score, defined for each variable (extracted bioactive principle), was calculated as a sum over its PLS components’ loadings weighted by the percentage of explained Y variance by each PLS component^45^.

## Results and discussion

### Composition of extracts

The extraction procedure involved small liquid: solid (w/w) ratios as to yield extracts more concentrated in bioactive compounds, eliminating the necessity of concentrating more dilute hydroethanolic solutions. The largest ratio employed was 6:1, significantly lower than the usual values reported in literature.

**Figure 1:**
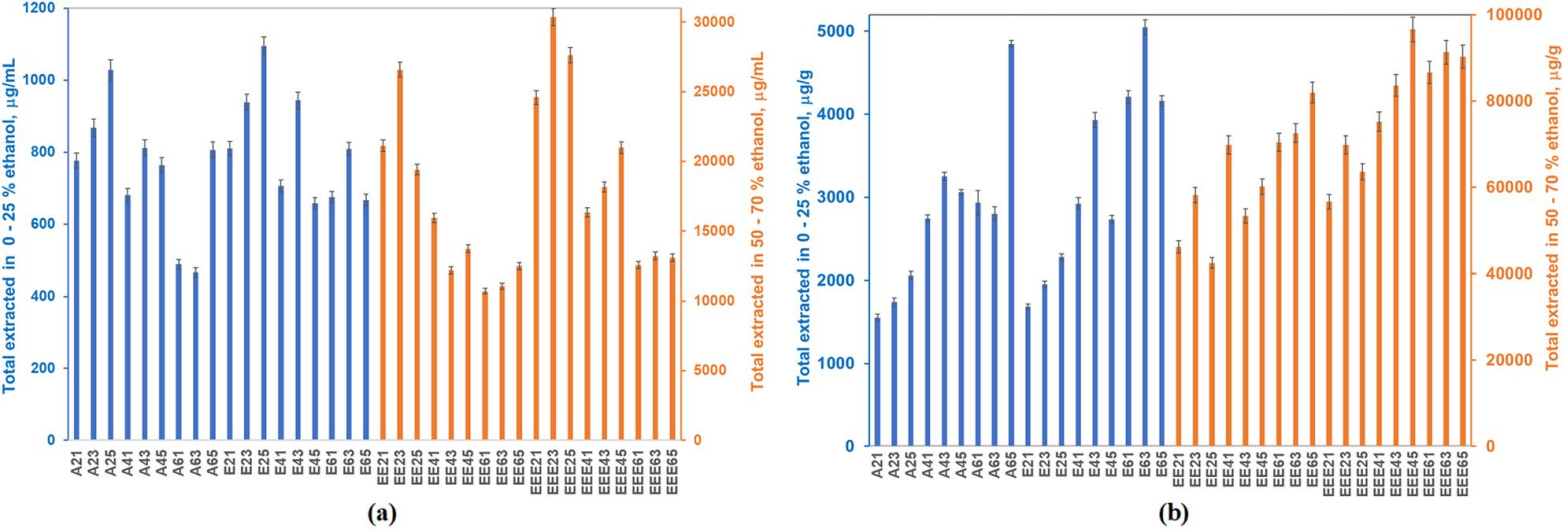
Total extracted polyphenolic acids and flavonoids expressed as (**a**) μg/mL and (**b**) μg/g propolis.

21 polyphenolics derivatives were quantified in the 36 extracts prepared and analysed, most also present in other papers on Romanian propolis^6,20,38–40,46^. Data collected, with relative standard deviations below 5%, identified bioactive principles with average content lower than 100 μg/g, below 1 mg/g, and a major group exceeding 1 mg/g. Total extracted phenolic acids and flavonoids quantified by the UHPLC-MS method are presented in Fig. 1.

Water and 25% ethanol solubilize on average 2.78 and 3.29 mg/g polyphenolics respectively, since the saturation concentrations are attained faster, and, thus, the mass driving force ceases to exist. The concentration of aqueous and ethanolic solutions increased linearly with the extraction time, for the 2:1 ratio, the ethanol influence being negligible. When the liquid phase volume increased, the concentrations of the extracts slightly decreased, almost always having a maximum after three days contact time. This could be an effect of the dichotomy between the availability of the active compounds from the solid matrix and the extracts’ degradation. In these cases, the presence of ethanol improved extraction, but not significantly for phenolic acids (Supplementary Figure S2a online), while significantly for flavonoids (Supplementary Figure S2b online).

The 50 and 70% ethanolic solutions extracted on average 58.03 and 78.4 mg/g bioactive components, depending on the liquid:solid ratio and the contact time; this is due to the raise of the saturation concentration, ensured by the presence of more ethanol molecules, likely to form molecular associations with the active compounds. The dichotomy between the active compounds’ availability and their stability is present again. Despite the reduced solid:liquid ratio employed in the present study, these values compare well to the 73.66 mg/g average for the total phenolic content reported by Marghitas et al.^39^.

Larger volumes of liquid, as in the 6:1 liquid:solid ratio, do not bring along higher concentrations of analytes in the extracts, as expected when a larger concentration gradient is present.

Representing on average 98.5% of the extracted compounds in water, phenolic acids are accompanied by 1.45% flavonoids, and 0.05% abscisic acid, the only terpenoid identified in this study. In 25% hydroalcoholic solutions, phenolic acids exceed 86%. In 50% ethanol, they represent less than 44% of the total, dropping to 40% in 70% ethanol.

The best extracted compound in water is *p*-coumaric acid (1.73 mg/g), while in 25% ethanol is ferulic acid (1.5 mg/g). The trend is less clear cut for 50% ethanol, since ferulic and *p*-coumaric acids part the supremacy with chrysin. 70% ethanol extracts chrysin most in all tests.

Chrysin, the reference flavonoid in poplar propolis, amounts on average to 12.4 mg/g in 50 -70% ethanol, a value larger than 1.6 mg/g propolis reported for the Romanian propolis^6^. A later focus on Transylvanian propolis reveals levels in the 0.55–3.91 mg/g range^39^, still lower than the content extracted from the Bihor county-originating product investigated here.

**Figure 2:**
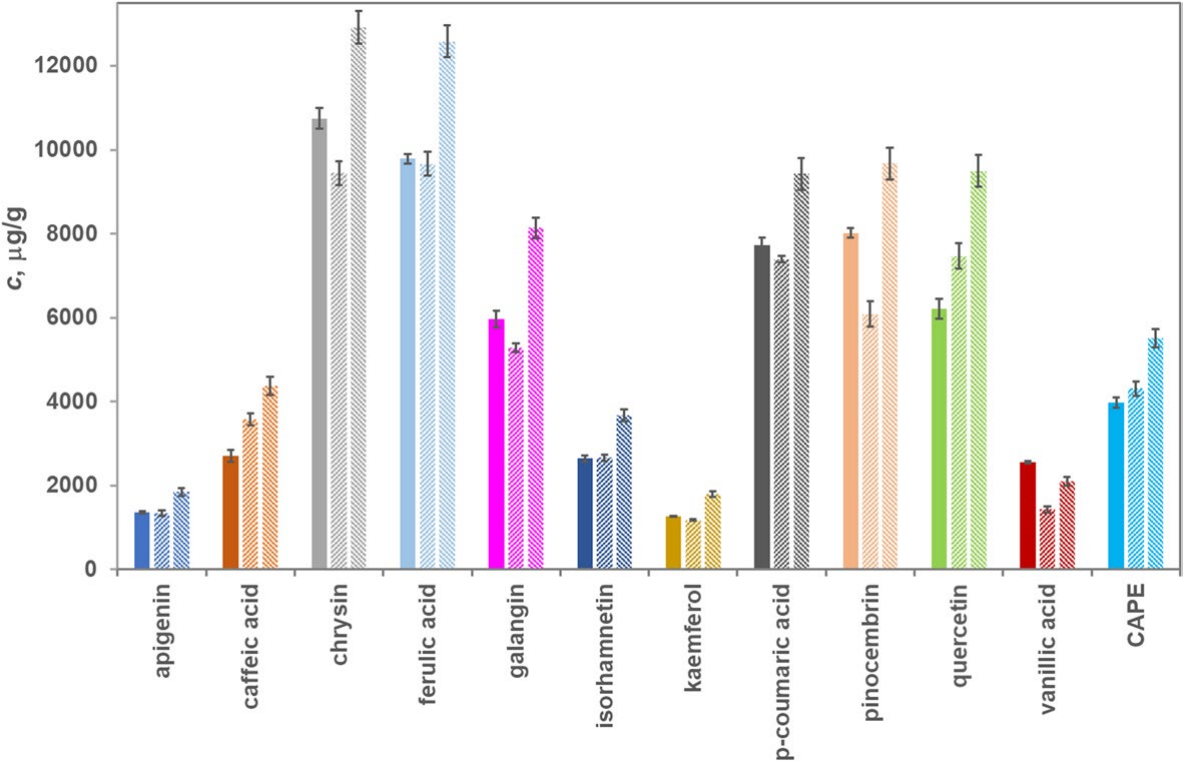
Polyphenolic derivatives composition pattern at different liquid : solid ratios in 50% ethanol (EE25–full colour, EE45–diagonal stripes upward, and EE65–diagonal stripes downward).

For a given solvent composition, the ratio between the major and minor extracted components was not significantly affected by the liquid: solid ratio, as shown in Fig. 2 and Supplementary Figures S3–S5, online.

CAPE, an established anti-tumoral agent^47,48^, was absent in water, present in traces in 25% ethanol, raising to 4.2 mg/g in 50% ethanol, and 4.9 mg/g in 70% ethanol. Some propolis specimens subjected to 100:1 (solid: liquid) extraction in 96% ethanol revealed levels in the 0.86–3.87 mg/g range^39^; local and seasonal variation in propolis composition may explain these differences.

Galangin and chrysin, also known for anti-tumoral effects^35^, represent 27.8% and 29.8% of extracted compounds in the 50% and 70% ethanol solution and in negligible amounts in the higher water-containing solvents. Supplementary Table S2 online presents the extract composition behaviour for all quantified phenolic acids and flavonoids, alongside their chemical structure.

Caffeic acid levels depend on the extraction conditions, as Coneac et al^46^ also noticed. They extracted 5.6 mg/g in cold 20% ethanol at 20:1 liquid: solid ratio. The present study reports only 0.7 mg/g in 25% ethanol at 6:1 liquid: solid ratio and 4.0 mg/g in 50% ethanol, a value closer to the published 6.34 mg/g in 60% ethanol^46^. It decreases in 96% ethanol to 2.45 mg/g^46^, a trend spotted for the 70% ethanol extracts in the present study (3.58 mg/g). This is in line with the 3.4 mg/g caffeic acid content found in some Italian propolis^5^.

Gatea et al.^38^ found higher levels of pinocembrin, CAPE, galangin, and chrysin in propolis from the neighbouring Arad County; only the *p*-coumaric acids levels are somewhat similar, 12.68 mg/g (Arad) to 9.6 mg/g (Bihor-present study). *p*-Coumaric acid was found in higher amounts in Suceava propolis^10^, accompanied by levels of galangin, pinocembrin, and quercetin comparable to the present study.

### Antioxidant effects variation

**Figure 3:**
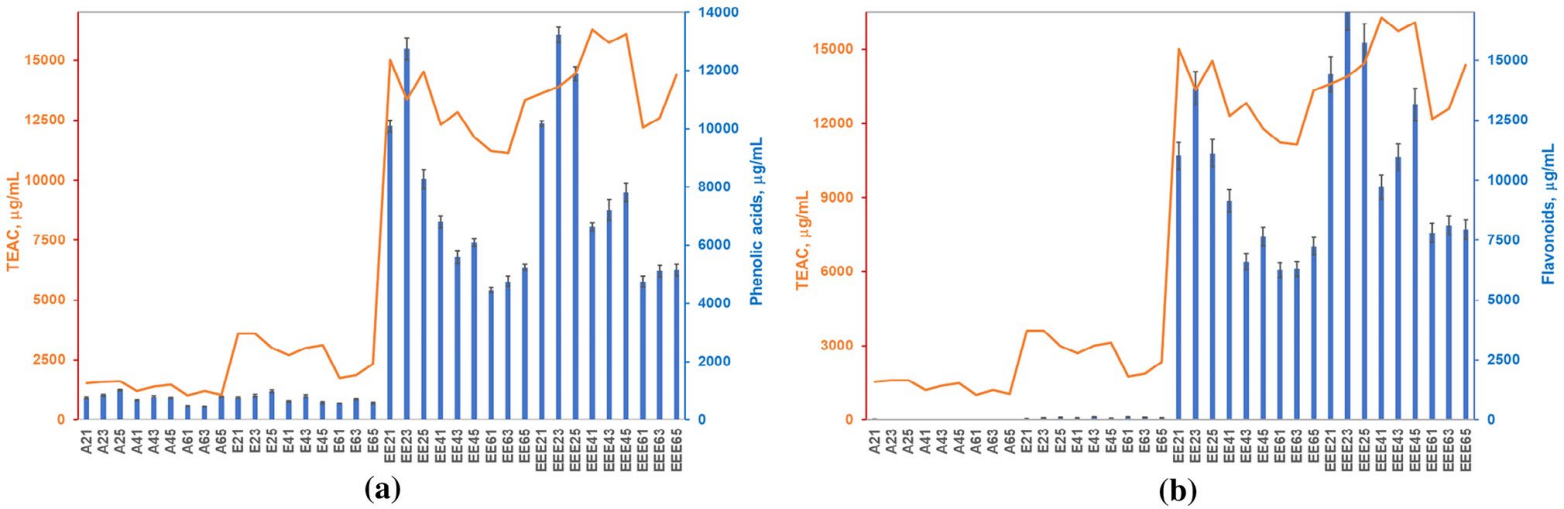
Antioxidant effects variation (ABTS assay) with phenolic acids (**a**) and flavonoids (**b**) distribution.

The antioxidant effects are imparted by all analytes quantified in this study. The registered antioxidant effects in aqueous type solvents, determined by ABTS quenching, are induced by acids and their esters, as flavonoids are present in negligible amounts (Fig. 3). Larger volumes of alcohol offer the premises of extracting more flavonoids, along larger amounts of some phenolic acids. The large increase in the antioxidant effects is attributable to the flavonoids (Fig. 3b).

After unit conversion, the antioxidant capacity average (11.47 mM TEAC/g in 70% ethanol) is in line with the 10.3 mM TEAC/g reported for samples collected in a neighbouring county in Romania, Arad, using ABTS method^38^.

**Figure 4:**
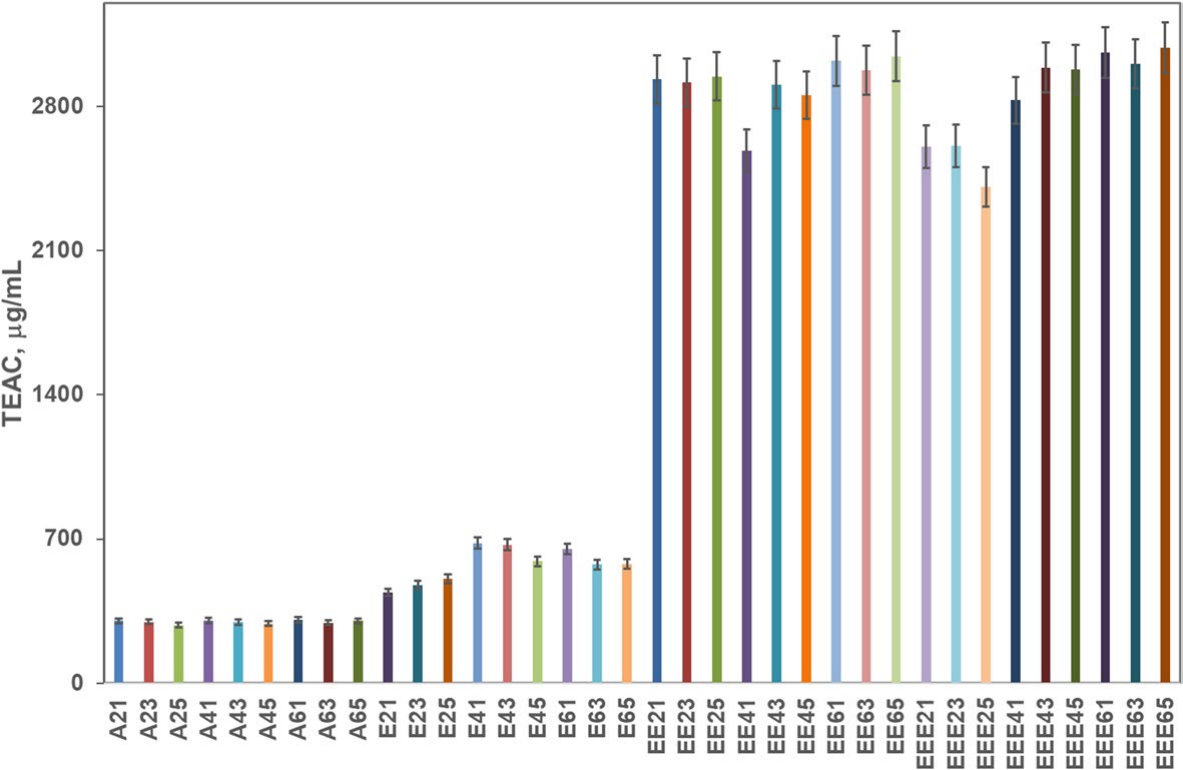
Antioxidant capacity of the propolis extracts evaluated with the DPPH method.

The DPPH assay signals similar antioxidant capacity values for 50 and 70% ethanolic solutions (Fig. 4). The 2,841 μg TEAC/mL average for 70% ethanol, at a 6:1 solid:liquid ratio converts into 2.44 mM TEAC/g, not so different from the 3.85 mM TEAC/g^38^ in Bihor and 1.05 mM TEAC/g^40^ in Cluj, Arad, and Alba neighbouring zones.

**Figure 5:**
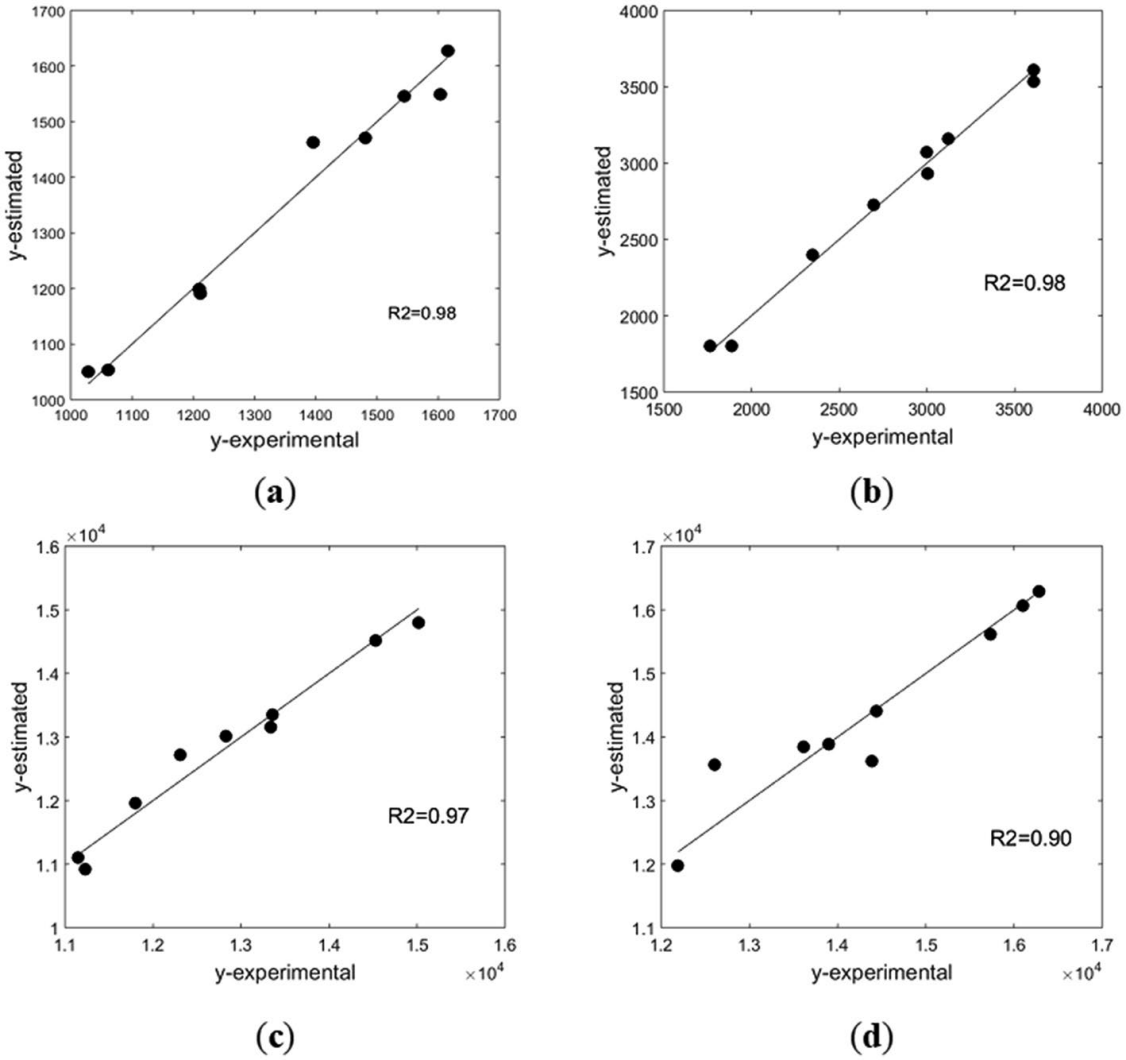
Model-experiment parity plots in various solvents: (**a**) water; (**b**) 25% ethanol; (**c**) 50% ethanol; (**d**) 70% ethanol.

PLS statistical modelling gave good correlations between the 21 compounds identified in the extracts and the antioxidant capacity of aqueous and ethanolic solvents, with the aid of six PLS components for water and 25% ethanol, and 7 PLS components for 50% and 70% ethanol (Fig. 5). Normalized values of compounds’ concentration in each individual extract were used, according to:

3 $$x_{i,j} = \frac{{c_{i,j} }}{{\max (c_{i,j} )}}$$

*j* standing for the extract, and *i* for the component. The antioxidant capacity, as determined by the ABTS method, was used in real units.

The compounds with the largest contribution in the correlation were selected for each specific solvent, based on their VIP score (Table 1 and Supplementary Figures S6–S9 online). The PLS analysis showed that in water the differentiation in composition according to operating conditions is mainly from phenolic acids (ferulic, caffeic, ellagic, and *p*-coumaric acids). Flavonoids with complex structures, such as chrysin, pinocembrin, and quercetin, credited with high antioxidant capacity^2^, are present in very small concentrations in water, but in 25% ethanol they have significant contributions in the differentiation of extract composition and antioxidant capacity. In more concentrated ethanolic solutions, the levels of chrysin, quercetin, pinocembrin or CAPE are sufficiently high, therefore their important contribution in the variation of antioxidant capacity is expected and confirmed.

**Table 1:** Significant chemical compounds in the PLS regression vector.

Solvent	Water extract	25% ethanolic extract	50% ethanolic extract	70% ethanolic extract
Compounds with VIP scores > 1	ferulic acid, caffeic acid, *p*-coumaric, acid ellagic, acid 4-hydroxybenzoic acid	caffeic acid, *p*-coumaric acid, ferulic acid, syringic acid, quercetin, pinocembrin, chrysin	caffeic acid, *p*-coumaric acid, ferulic acid, vanillic acid, CAPE, quercetin, chrysin, kaempferol galangin	*p*-coumaric acid, 4-hydroxybenzoic acid, ferulic acid, quercetin, CAPE, kaempferol, galangin, pinocembrin, apigenin

**Figure 6:**
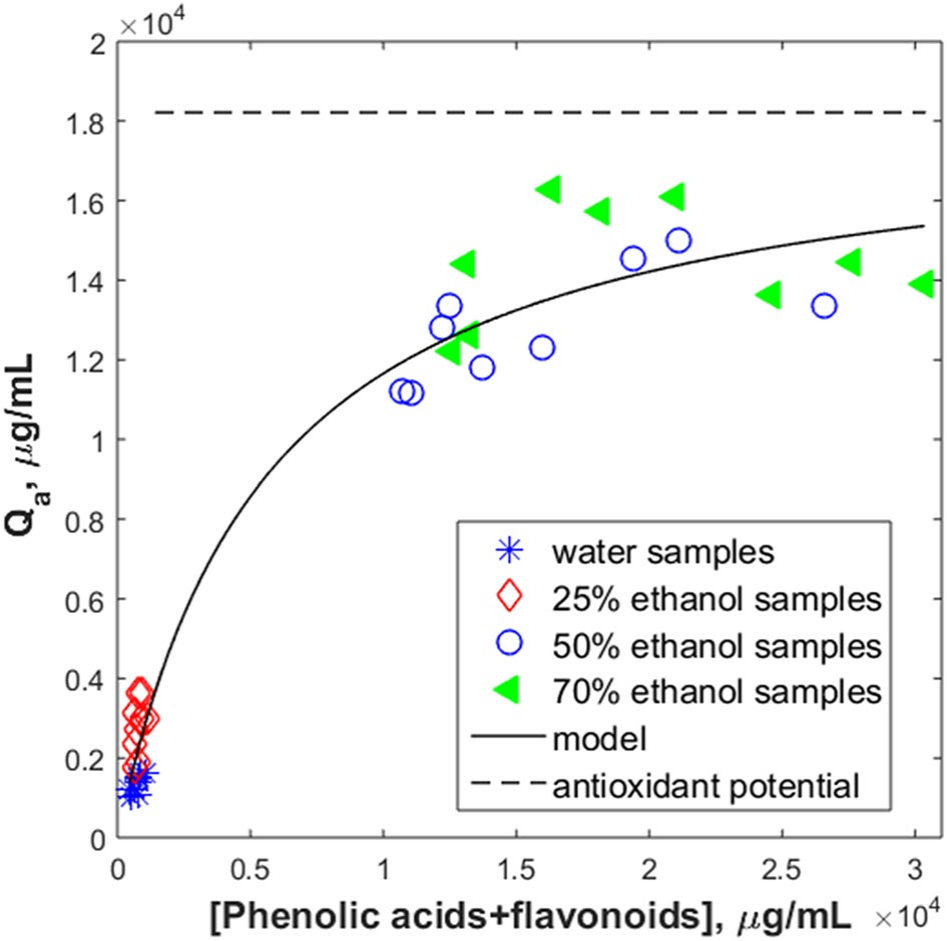
Antioxidant capacity as function of samples total polyphenolic derivatives content.

The saturation model (1) was tested first for the dependency of extracts antioxidant capacity on the total phenolic acids and flavonoids levels. The regression analysis over the experimental data was carried out in Matlab® R2015 (MathWorks, Natick, MA), using genetic algorithms, implemented as *ga* function, for the minimization of objective function, Eq. (2). Figure 6 reflects how the model fits experimental data. The coefficient of determination, *R*^2^, is 0.96 for *K*_*max*_ = 18,216 μg TEAC/mL extract, and *K*_*c*_ = 5632 μg polyphenolic derivatives/mL extract. As the maximum antioxidant capacity registered experimentally is 16,287 μg TEAC/mL extract, the *K*_*max*_ value shows a possible increase of antioxidant capacity by increasing the polyphenolic derivatives content in the extracts.

The antioxidant capacity increases with the growth of total phenolics concentration, with a steep increase in the 25–50% ethanol region. This may be due to the increased contribution of flavonoids beginning with 50% ethanol. The model slope decreases drastically for 50–70% ethanol, reflecting the experimental values’ oscillations around 15,000 μg TEAC/mL. According to this model, the increase above 50% ethanol content in the extractant does not bring important changes in the antioxidant capacity. This agrees with the propolis-selling beekeepers’ practice; they use at most 40% alcoholic solutions (mainly 30%) to extract bioactive principles from propolis. Still, some intensification techniques could be beneficial, to increase the polyphenols and flavonoids content, thus, the antioxidant capacity.

The antioxidant capacity of the extracts was also corelated with the concentration of some components which: (a) were present in larger amounts in the extracts, (b) proved to have important contribution in the PLS model (Supplementary Table S3 and Figure S10, online).

Analysis of the calculated model parameters might bring further insight in the correlation between structure and antioxidant contribution of a given extract. As *K*_*c*_ reflects the value of the component concentration for which the extract antioxidant capacity is half of the antioxidant potential, when individual components are analysed, this value should be related to the maximum concentration revealed in the studied extracts, *c*_max_. The *K*_*c*_/*c*_max_ ratios are 0.48 for *p*-coumaric acid, 0.30 for ferulic acid, and 0.10 for caffeic acid; for quercetin this ratio is 0.06, proving that phenolic acids must reach higher concentrations than flavonoids to ensure similar increased antioxidant capacity.

Higher *K*_*max*_*/K*_*c*_ ratio could be a measure of a more important antioxidant contribution of the component. These ratios are 10 for *p*-coumaric acid, 14 for ferulic acid, and 29 for caffeic acid, while for flavonoids these ratios are 220 for pinocembrin, and 436 for galangin, signalling the more important contribution of flavonoids in the antioxidant capacity of propolis extract. These observations are in good agreement with theoretical considerations^49,50^, based on molecular structure, which indicate an increase of antioxidant capacity of phenolic acids in the order:

$$p - {\text{coumaric}}\;{\text{acid}} < {\text{ferulic}}\;{\text{acid}} < {\text{caffeic}}\;{\text{acid}}.$$

On the other hand, in flavonoids, multiple hydroxyl groups and their relative positions enhance the antioxidant capacity^50,51^, as is the case of quercetin, galangin, and kaempferol, also identified in the present study as important components in the definition of antioxidant capacity.

### Antimicrobial activity quantification

Table 2 presents the antimicrobial activity of the studied propolis extracts. Aqueous propolis extracts show a constant and substantial bactericidal activity against G −, but not so high against G + (Supplementary Figure S11 online). Higher alcohol containing extracts are more active against the G + *B. subtilis* bacteria. The antifungal action against *C. albicans* is moderate and rather similar for all tested extracts^6^. Aqueous extracts, containing mainly phenolic acids, proved to act on *E. coli* and *C. Albicans*. The extract with best antibacterial effect on *E. coli* is A65, with higher liquid:solid ratio during extraction. Antimicrobial activity is the effect of the significant concentrations of *p*-coumaric acid, found in all aqueous propolis extracts. The higher activity of the more diluted extracts might be explained by the increase of *p*-coumaric concentration in the total polyphenolics content visible for 3- and 5-day extraction time (A23–0.334 *verus* A63–0.377). A dual mechanism^52^ explains its bactericidal activity: disruption of bacterial cell’s outer membranes and inhibition of cellular functions through binding to bacterial genomic DNA, ultimately leading to cell death. This activity is higher for G − bacteria^53^ (diderm bacteria with outer and inner membranes), much more susceptible to *p*-coumaric acid, due to their content of lipopolysaccharides, contrary to G + (monoderm bacteria), with lower liposaccharides content. The PLS analysis also identified the important contribution of *p*-coumaric acid to the antioxidant capacity.

**Table 2:** The antimicrobial activity of propolis extracts.

Extract	*E. coli*		*B. subtilis*		*C. albicans*	
	Inhibition zone, mm	MIC, μg/mL	Inhibition zone, mm	MIC, μg/mL	Inhibition zone, mm	MIC, μg/mL
A21	0.50^a^	< 1663	1.00^a^	4990	1.33 ± 0.47	< 832
A41	1.00^a^		*na*		2.33 ± 0.94	
A61	2.00^a^		*na*		2.67 ± 0.47	
A23	0.50^a^	< 832	0.67 ± 0.24	2495	1.00 ± 0.82	< 832
A43	1.00^a^		0.50^a^		2.00 ± 0.82	
A63	2.33 ± 0.47		*na*		1.33 ± 0.47	
A25	*na*	<832	0.50^a^	< 1633	2.01	< 832
A45	1.00^a^		0.50^a^		2.00 ± 1.00	
A65	2.67 ± 0.47		0.508		2.67 ± 0.47	
E21	*na*	1610	1.67 ± 0.47	< 805	*na*	1610
E41	*na*		2.00^a^		*na*	
E61	1.33 ± 0.7		2.00^a^		1.67 ± 0.47	
E23	*na*	1610	1.33 ± 0.47	< 805	na	2415
E43	*na*		2.00^a^		0.33 ± 0.12	
E63	1.67 ± 0.47		2.67 ± 0.47		*na*	
E25	*na*	1610	*na*	1208	1.00 ± 0.94	805
E45	*na*		2.67 ± 0.47		na	
E65	1.00^a^		*na*		0.83 ± 0.5	
EE21	1.00^a^	< 1537	2.00	< 768.5	*na*	2305
EE41	1.00^a^		4.67 ± 0.47		1.00^a^	
EE61	1.33 ± 0.47		4.33 ± 0.47		1.00^a^	
EE23	1.00 ± 0.41	< 1537	3.67 ± 1.25	< 769	0.67 ± 0.14	769
EE43	1.00^a^		1.67 ± 0.47		1.33 ± 0.47	
EE63	1.67 ± 0.47		2.33 ± 1.25		1.33 ± 0.47	
EE25	*na*	< 769	2.33 ± 1.25	< 76	*na*	1153
EE45	2.00^a^		4.00^a^		1.67 ± 1.25	
EE65	1.33 ± 0.47		3.00 ± 0.82		*na*	
EEE21	0.06^a^	< 1440	0.33 ± 0.27	< 720	*na*	1440
EEE41	1.94 ± 0.63		2.78 ± 1.16		*na*	
EEE61	1.61 ± 0.68		3.22 ± 0.16		0.33 ± 0.02	
EEE23	*na*	< 720	2.00 ± 0.47	< 720	*na*	1080
EEE43	1.72 ± 0.72		4.67 ± 0.47		1.00 ± 0.16	
EEE63	3.39 ± 0.47		3.78 ± 0.42		1.22 ± 0.31	
EEE25	*na*	1080	1.56 ± 0.31	< 720	*na*	< 2160
EEE45	1.50 ± 0.54		3.33 ± 0.98		0.89 ± 0.16	
EEE65	1.83 ± 0.57		2.22 ± 0.16		0.78 ± 0.16	

*na* non active (no areas of inhibition reported).

^a^identical replicates.

*E. coli* Escherichia coli.

*B. subtilis* Bacillus subtilis spizizenii nakamura.

*C. albicans* Candida albicans.

The antifungal effect of *p*-coumaric acid on *Candida albicans* is the result of its action upon the cell membrane and ergosterols synthesis^54^. Banskota *et al*^27^ were among the first to link the anti-fungal activity of ethanolic propolis extracts to pinocembrin, *p*-coumaric, and caffeic acids, and little other information on antifungal effects of water extracts is available. Even if the present aqueous extracts do not contain flavonoids, their proven anti-fungal effects are to be related to the presence of caffeic and *p*-coumaric acids. Data in Table 2 proves that aqueous extracts are more effective for G − and unicellular fungi (MIC < 831.7 μg/mL) compared to the G + (< 1633.3 μg/mL).

Phenolics profile of 25% ethanol extracts is like the aqueous ones, except for the quercetin content. Quercetin levels could explain the increase in the antimicrobial effect upon the G + *B. subtilis* (MIC < 805 μg/mL), along with the demonstrated significant contribution to the antioxidant capacity.

The 50% ethanolic extracts contain, in addition to phenolic acids, large quantities of chrysin and galangin, flavonoids with already proven high antioxidant contribution. The high flavonoids levels seem responsible for the better reaction towards *B. subtilis*, in line with the reports of Mocanu *et al*^55^.

Chrysin and galangin are proven to inhibit the growth of *Candida spp*., by repressing the biofilm formation^54,56,57^. The present experimental data indicates that the optimal concentration of phenolic acids and flavonoids is reached after 3 days extraction in 50% ethanol (Supplementary Figure S11, online). All extracts, regardless the liquid:solid ratio, exhibit antimicrobial activity, the most efficient extract being EE45. 50% ethanolic extracts showed the lowest inhibitory concentration for G − after 5 days, 768.5 μg/mL.

More ethanol in the extracting solvent (70%) does not bring further increase, neither in the antimicrobial, nor in the anti-fungal activity. Quercetin, chrysin, and pinocembrin, in the company of comparable levels of ferulic and *p*-coumaric acids enable a synergetic antimicrobial activity. The results obtained indicate a more pronounced antibacterial action against G + than on G − bacteria^40,58,59^, probably because quercetin contribution is hindered by co-extracted compounds. EEE43 is more efficient for *B. subtilis*., while EEE63 for *E. coli*.

The antimicrobial and anti-fungal investigation of the propolis extracts demonstrated that 70% ethanol does not bring advantages compared to the 50% ethanol extracts, a conclusion similar in terms of antioxidant capacity.

Antimicrobial activity enhances the results of antioxidant capacity, proving the tight bound and effect displayed by the composition of different types of Romanian propolis extracts.

## Conclusions

The profile of phenolic acids and flavonoids extracted from propolis proved determinant for both the antioxidant and antimicrobial activity. Its characteristics are regulated by operational parameters. Contribution of phenolic acids to antioxidant capacity is present in all studied extracts. Higher ethanol concentration asymptotically favoured the extraction of flavonoids, which, in turn, increased free radicals scavenging ability and activity against the G + and yeast strains used.

Bioactive principles in high concentrations are responsible for the registered antioxidant capacity in 50 and 70% ethanolic extracts and the inhibitory effect on the growth of all tested strains, highest in 50% ethanolic extracts.

Statistical modelling results gave important insight into the relation between the polyphenolic profile and antioxidant capacity, in good agreement with theoretical considerations.

## Supplementary Information

Supplementary Information.
