# Supplementary material for: Polyphenolics profile effects upon the antioxidant and antimicrobial activity of propolis extracts
Source: Sci Rep. 2021 Oct 11;11:20113. doi: 10.1038/s41598-021-97130-9 (PMC8505647; doi:10.1038/s41598-021-97130-9)
Supplement: Supplementary file 1 — Supplementary Information. [file 41598_2021_97130_MOESM1_ESM.docx]

**SUPPLEMENTARY INFORMATION**

**POLYPHENOLICS PROFILE EFFECTS UPON THE ANTIOXIDANT AND ANTIMICROBIAL ACTIVITY OF PROPOLIS EXTRACTS**

*Mădălina Maria Nichitoi,^1^ Ana Maria Josceanu,^2*^ Raluca Daniela Isopescu,^3^ Gabriela Olimpia Isopencu,^3^* Irina Geana,^4^ Elisabeta Ciucure^4^, Vasile Lavric*,*3*

^1^ University Politehnica of Bucharest, Doctoral School “Applied Chemistry and Materials Science”; [maria.nichitoi@yahoo.com](mailto:maria.nichitoi@yahoo.com)

^2^ University Politehnica of Bucharest, Department of Analytical Chemistry and Environmental Engineering; [a_josceanu@chim.upb.ro](mailto:a_josceanu@chim.upb.ro)

^3^ University Politehnica of Bucharest, Department of Chemical and Biochemical Engineering; [r_isopescu@chim.upb.ro](mailto:r_isopescu@chim.upb.ro) (RDI); [g_isopencu@chim.upb.ro](mailto:g_isopencu@chim.upb.ro) (GOI); [lavric.vasile@gmail.com](mailto:lavric.vasile@gmail.com) (VL);

^4^ National Research and Development Institute for Cryogenics and Isotopic Technologies – ICSI; [irina.geana@icsi.ro](mailto:irina.geana@icsi.ro) (IG); [corina.ciucure@icsi.ro](mailto:corina.ciucure@icsi.ro) (CTC)

* Correspondence: [a_josceanu@chim.upb.ro](mailto:a_josceanu@chim.upb.ro); g_isopencu@chim.upb.ro; Tel.: AMJ +40744543020, GOI +40 21 402 39 69

**Table S1.** Calibration data for the phenolic acids and flavonoids profiling

| **No.** | **Compound** | **Retention time**  **[min]** | **Accurate mass [M‐H]^−^** | **Mass fragments** | **Calibration curve parameters** | | | | **Performance characteristics** | |
| --- | --- | --- | --- | --- | --- | --- | --- | --- | --- | --- |
|  |  |  |  |  | **10^-5^ ×slope** | **10^-3^ ×*s*_slope_** | **R^2^** | **10^-7^ ×*s*_y/x_** | **LOD [μg/mL]** | **LOQ [μg/mL]** |
| **1** | Gallic acid | 0.68 | 169.0133 | 125.0231 | 5.29 | 1.08·10^4^ | 0.9971 | 3.02 | 0.188 | 0.571 |
| **2** | 3,4-Dihydroxybenzoic acid | 1.59 | 153.0183 | 109.0281 | 5.74 | 1.08·10^4^ | 0.9975 | 3.00 | 0.173 | 0.523 |
| **3** | 4-Hydroxybenzoic acid | 5.40 | 137.0232 | 93.0331 | 1.83 | 4.60 | 0.9956 | 1.28 | 0.231 | 0.699 |
| **4** | Catechin | 6.67 | 289.0719 | 109.0282, 125.0232, 137.0232, 151.0390, 203.0708, 245.0817 | 3.35 | 7.85 | 0.9962 | 2.19 | 0.216 | 0.653 |
| **5** | Chlorogenic acid | 7.55 | 353.0879 | 191.0553 | 2.24 | 4.11 | 0.9976 | 1.15 | 0.169 | 0.512 |
| **6** | Caffeic acid | 7.69 | 179.0342 | 135.044 | 9.03 | 19.3 | 0.9968 | 5.37 | 0.197 | 0.596 |
| **7** | Epi-catechin | 7.98 | 289.0719 | 109.0282, 125.0232, 137.0232, 151.0390, 203.0708, 245.0817 | 4.35 | 8.76 | 0.9972 | 2.44 | 0.185 | 0.561 |
| **8** | Syringic acid | 8.03 | 197.0450 | 182.0212, 166.9976, 153.0547, 138.0311, 123.0075 | 0.24 | 0.417 | 0.9982 | 0.090 | 0.124 | 0.376 |
| **9** | Vanillic acid | 8.31 | 167.0343 | 152.0105,124.0154, 111.0075,139.0025, 95.0125 | 0.0389 | 0.0818 | 0.9978 | 0.018 | 0.151 | 0.456 |
| **10** | *p*-Coumaric acid | 8.59 | 163.0392 | 119.0489 | 8.61 | 15.4 | 0.9981 | 3.34 | 0.128 | 0.388 |
| **11** | *t*-Ferulic acid | 8.83 | 193.0500 | 178.0262, 134.0361 | 2.16 | 3.40 | 0.9983 | 0.947 | 0.145 | 0.439 |
| **12** | Ellagic acid | 9.66 | 300.9990 | 300.9990 | 2.10 | 6.10 | 0.9941 | 1.70 | 0.267 | 0.810 |
| **13** | Abscisic acid | 10.04 | 263.1288 | 179.9803, 191.9454 | 6.61 | 10.4 | 0.9985 | 2.25 | 0.112 | 0.340 |
| **14** | Quercetin | 10.74 | 301.0356 | 151.0226, 178.9977, 121.0282, 107.0125 | 0.692 | 1.09 | 0.9983 | 0.304 | 0.145 | 0.439 |
| **15** | Kaempferol | 11.62 | 285.0406 | 151.0389, 117.0180 | 7.94 | 19.6 | 0.9958 | 5.46 | 0.227 | 0.687 |
| **16** | Isorhamnetin | 11.80 | 315.0512 | 300.0276 | 2.34 | 3.72 | 0.9985 | 0.81 | 0.114 | 0.344 |
| **17** | Apigenin | 11.86 | 269.0457 | 117.0333, 151.0027, 107.0126 | 19.6 | 86.5 | 0.9922 | 8.15 | 0.138 | 0.417 |
| **18** | Pinocembrin | 12.70 | 255.0663 | 213.0551, 151.0026, 107.0125 | 20.9 | 103 | 0.9903 | 16.2 | 0.256 | 0.775 |
| **19** | CAPE | 13.30 | 283.0975 | 174.9551, 112.9843 | 11.4 | 17.2 | 0.9880 | 23.2 | 0.140 | 0.399 |
| **20** | Chrysin | 13.52 | 253.0506 | 143.0491, 145.0284, 107.0125, 209.0603, 63.0226, 65.0019 | 19.9 | 103 | 0.9843 | 28.7 | 0.474 | 1.437 |
| **21** | Galangin | 13.77 | 269.0458 | 169.0650, 143.0491 | 19.9 | 43.9 | 0.9971 | 9.51 | 0.158 | 0.478 |

| **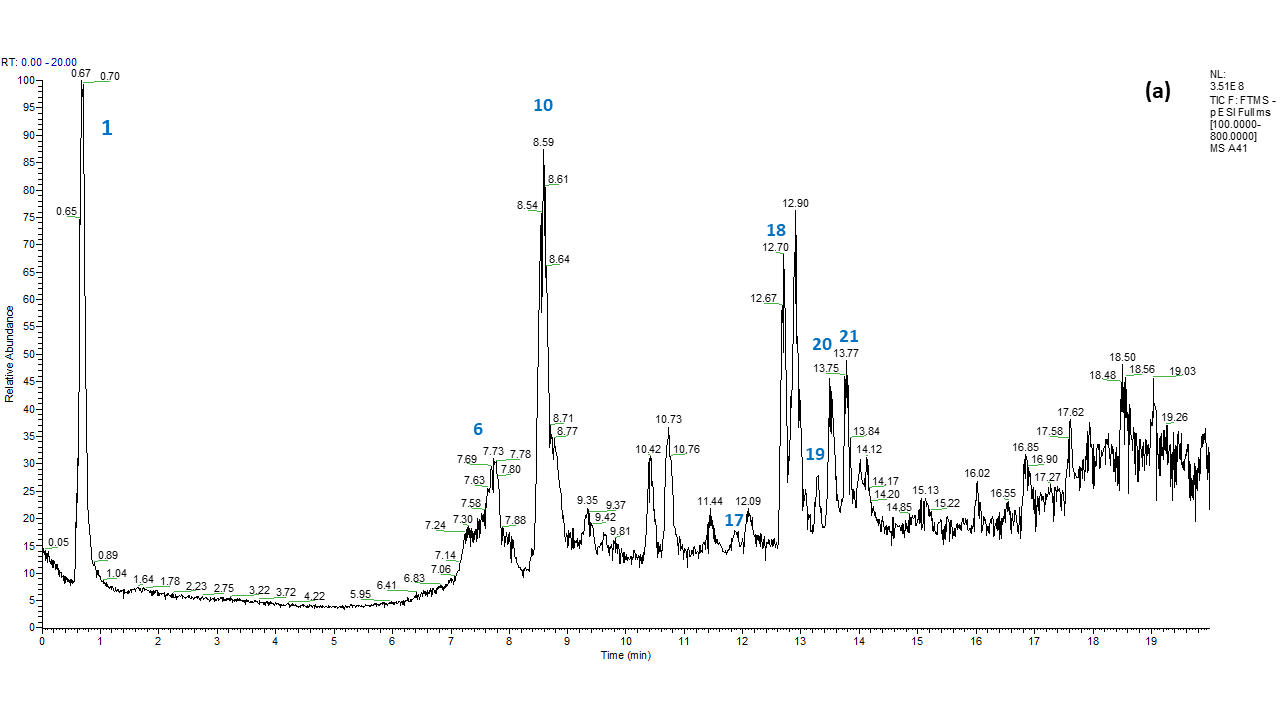** |
| --- |
| **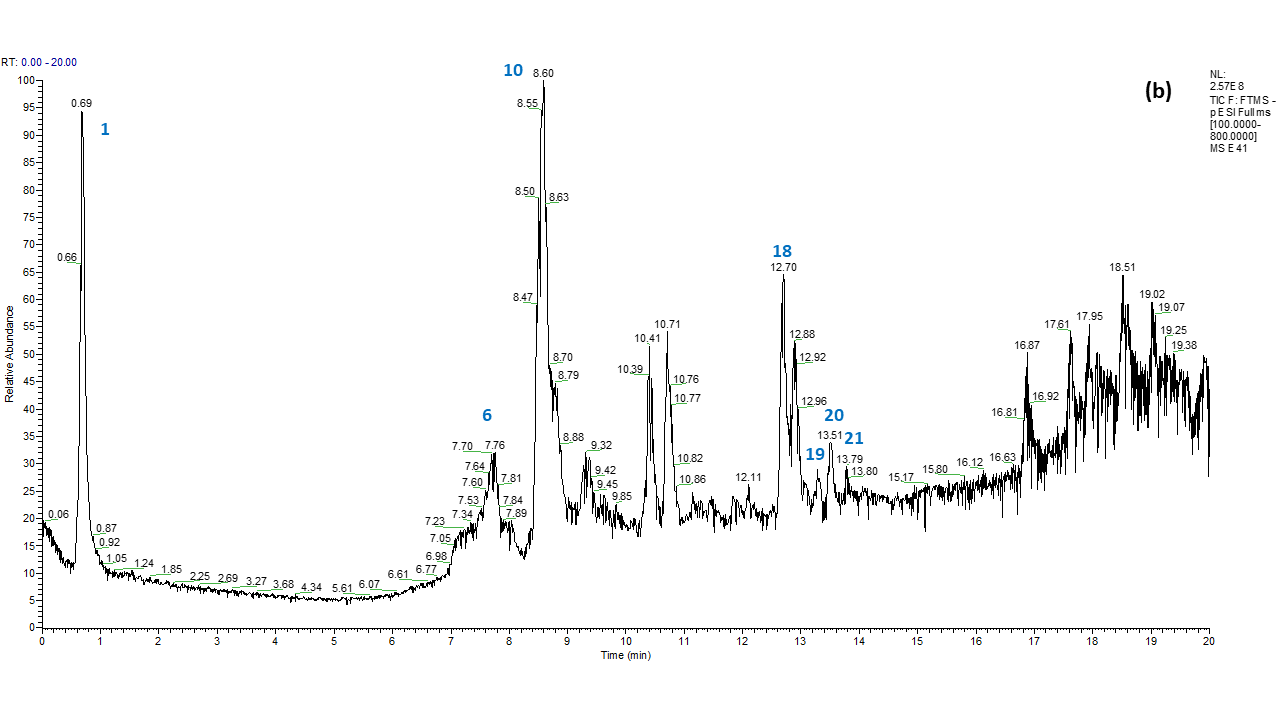** |
| **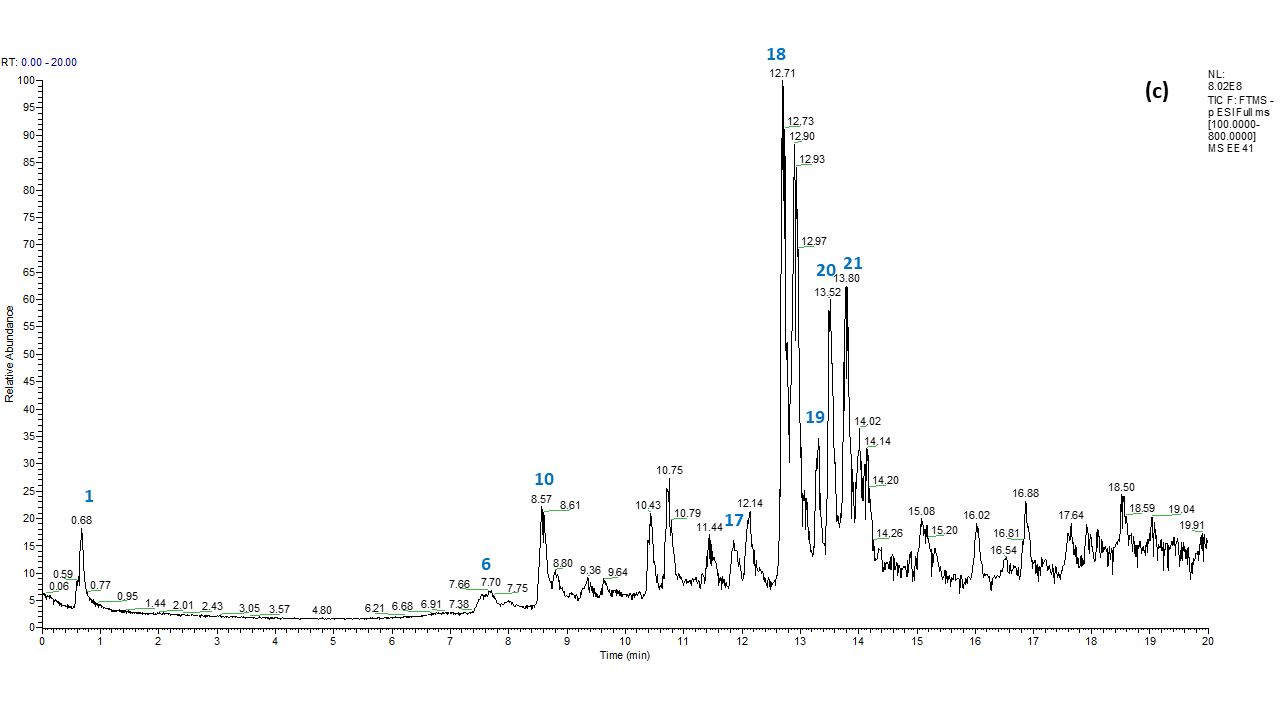** |
| **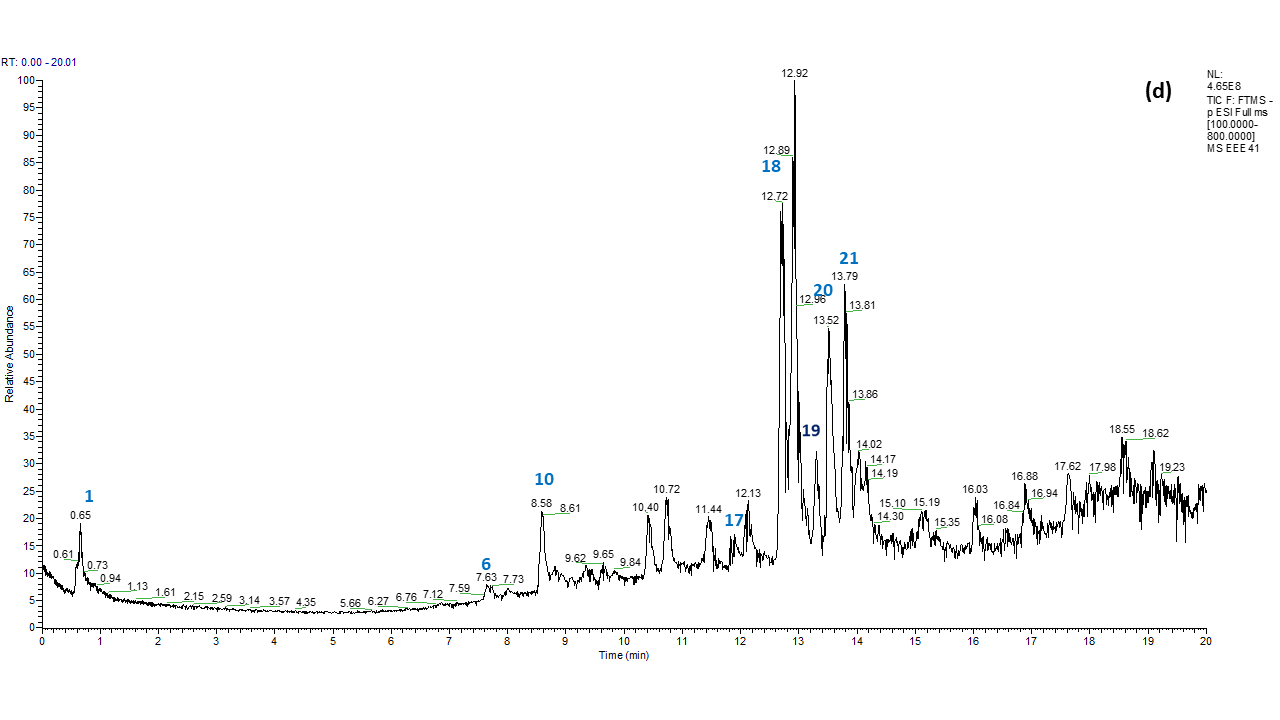** |

**Figure S1.** Base peak chromatograms of polyphenolic compounds in propolis extracts: (a) A41, (b) E41, (c) EE41 and (d) EEE41. The numbers identify analytes according to the numbering in Table S1.

**Table S2.** Extraction details by solvent and type of compound

| **Compound** | **Chemical structure** | **Amount extracted, μg/g propolis** | | | | **Comments** |
| --- | --- | --- | --- | --- | --- | --- |
|  |  | Water | 25 % ethanol | 50 % ethanol | 70 % ethanol |  |
| 3,4-Dihydroxybenzoic acid |  | < 2.3 | 0 – 2.23 | 0 – 11.6 | max 46.4 | always bellow 50 μg/g, regardless the solvent and liquid:solid ratio; maximum value reached in EEE63 |
| Abscisic acid |  | 0.79-2.52 | 0.74 - 2.14 | min 9.32 | max 15.54 | maximum value is reached in EEE45 |
| Catechin |  | < LOQ | 0.02 - 0.08 | 0 – 0.1 | max 0.20 | extraction is not favoured by the most concentrated conditions; maximum values reached in EEE63 |
| Chlorogenic acid |  | 1.32 | 0.88 | > 4.67 | < 14 | 2.1 μg/g (A65) and 14 μg/g (EEE63) |
| epi-Catechin |  | 0.15-0.29 | 0.16-0.27 | 0.50-2.15 | 0.25 – 2.47 | maximum values recorded for EE63 and EEE41 |
| Gallic acid |  | 3.16 – 36.3 | 2.32 – 5.38 | > 4.01 | >10.5 | a maximum of 36.3 μg/g for sample A65 and 17.2 μg/g for EEE61 |
| 4-Hydroxybenzoic acid |  | < 17 | 0 – 5.38 | 29.66 – 140.7 | 43.8 – 211.8 | EEE63 presents the maximum amount |
| Ellagic acid |  | 16.7-12.4 | 5.5 - 10.6 | > 43.4 | >112 | maximum value for EEE63 (156.9 μg/g) |
| Apigenin |  | 1.36 | 20.84 | 1426 | 1711 | maximum levels in EE65 (1.85 mg/g), even larger in EEE61 (2.0 mg/g) |
| Kaempferol |  | 1.30 | 17.39 | 1395 | 1477 | 1.79 mg/g for EE65  1.73 mg/g for EEE45 |
| Syringic acid |  | 15.04 | 194.96 | 216.14 | 235.7 | the only phenolic acid in the middle group;  maximum value reached in EE23 (453 μg/g) |
| Vanillic acid |  | 110 | 110.54 | 1780.3 | 2681.6 | the first in the category of major analytes in the analysed extracts;  maximum value in EEE65 (3.35 mg/g) |
| Isorhamnetin |  | 1.88 | 36.5 | 2641 | 3019 | maximum level in EE65 (3.7 mg/g) |
| Caffeic acid |  | 774 | 552 | 4036 | 3132 | maximum value in EE23 (5.1 mg/g) |
| Caffeic acid phenyl ester (CAPE) | 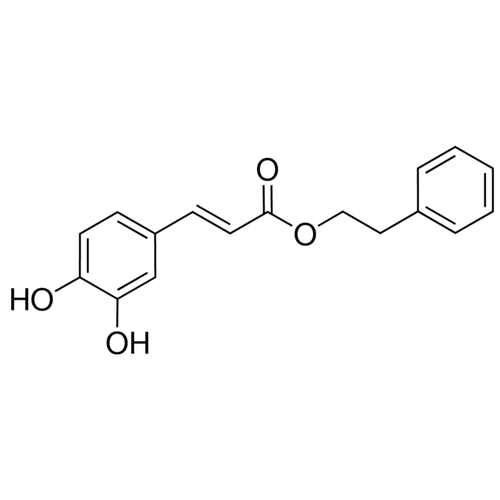 | <LOD | 28 | 4123 | 4876 | highest level in EEE61 (6.03 mg/g) |
| Galangin |  | 2.49 | 19.6 | 6248 | 7248 | highest level is attained for EEE63 (8.3 mg/g), the highest value of all identified compounds |
| Quercetin |  | 11.85 | 207.5 | 6524 | 8291 | the maximum value is found for EEE45 (8.5 mg/g) |
| Pinocembrin |  | 14.2 | 44.5 | 6468 | 10666 | the highest level is attained for EEE63 sample (13.6 mg/g) |
| *p*-Coumaric acid |  | 971 | 905 | 8044 | 8939 | a maximum for EEE45 (10.37 mg/g);  the lowest value recorded for A21 (538 μg/g). |
| *t*-Ferulic acid |  | 823 | 1013 | 8600 | 11250 | the highest value was recorded for sample EEE45 (13.153 mg/g); the lowest values was A23 (461 μg/g) |
| Chrysin |  | 6.6 | 45.9 | 9850 | 14900 | the largest value of an extracted analyte in this study is characteristic to EEE63 sample (18.86 mg/g) |


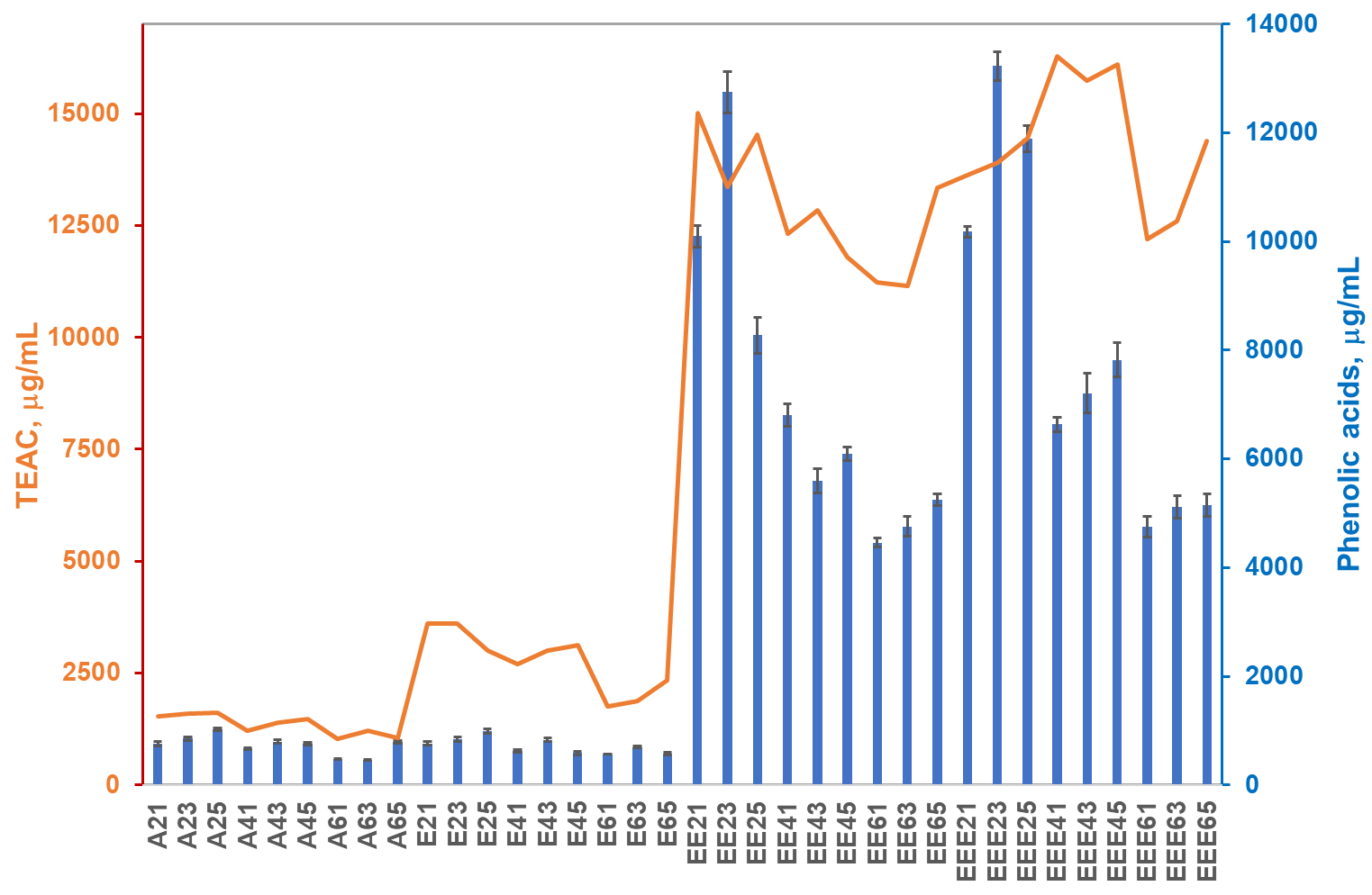


(a)


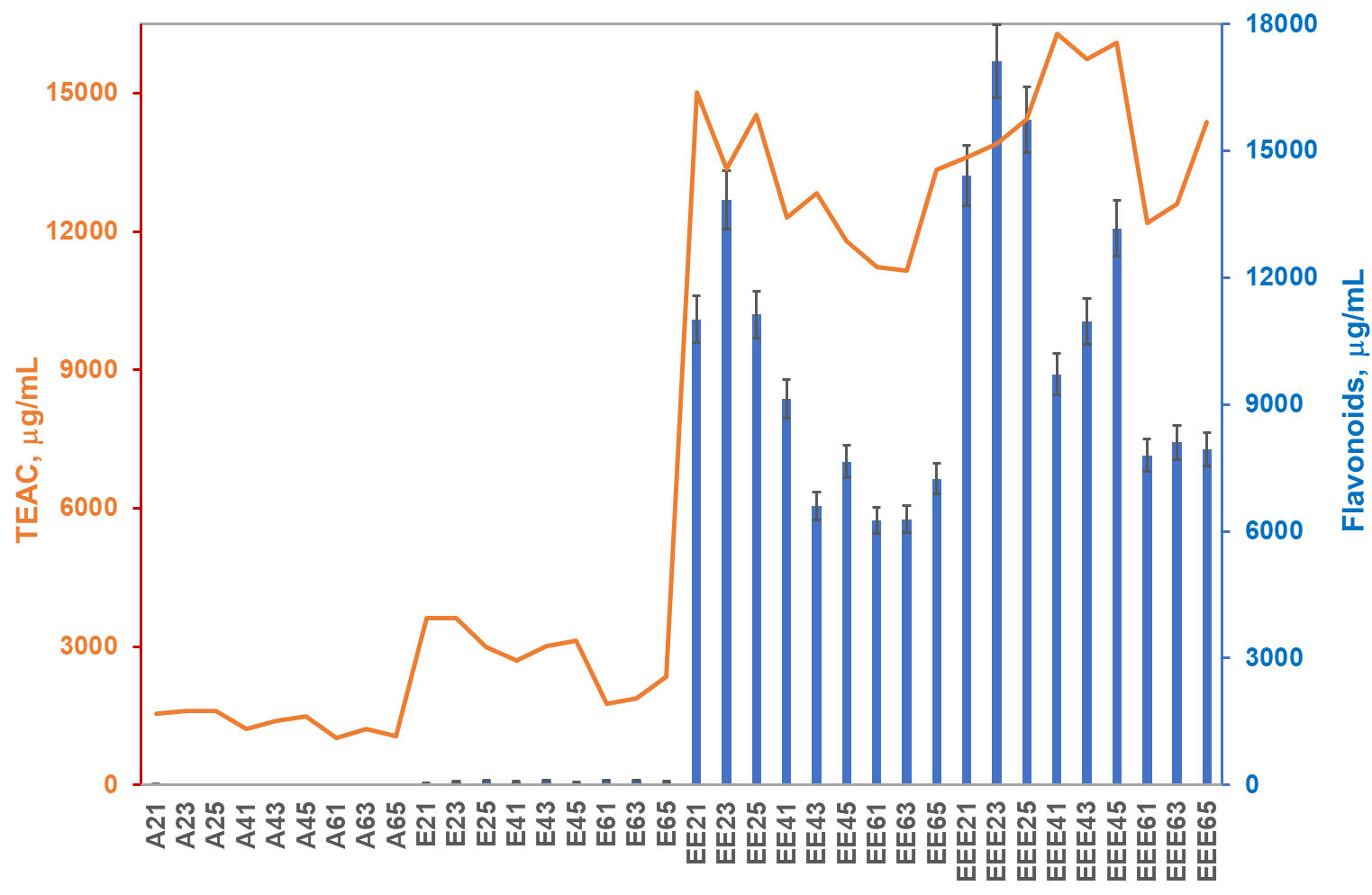


(b)

**Figure S2.** Variation of phenolic acids (a) and flavonoids (b) levels extracted in the tested solvents


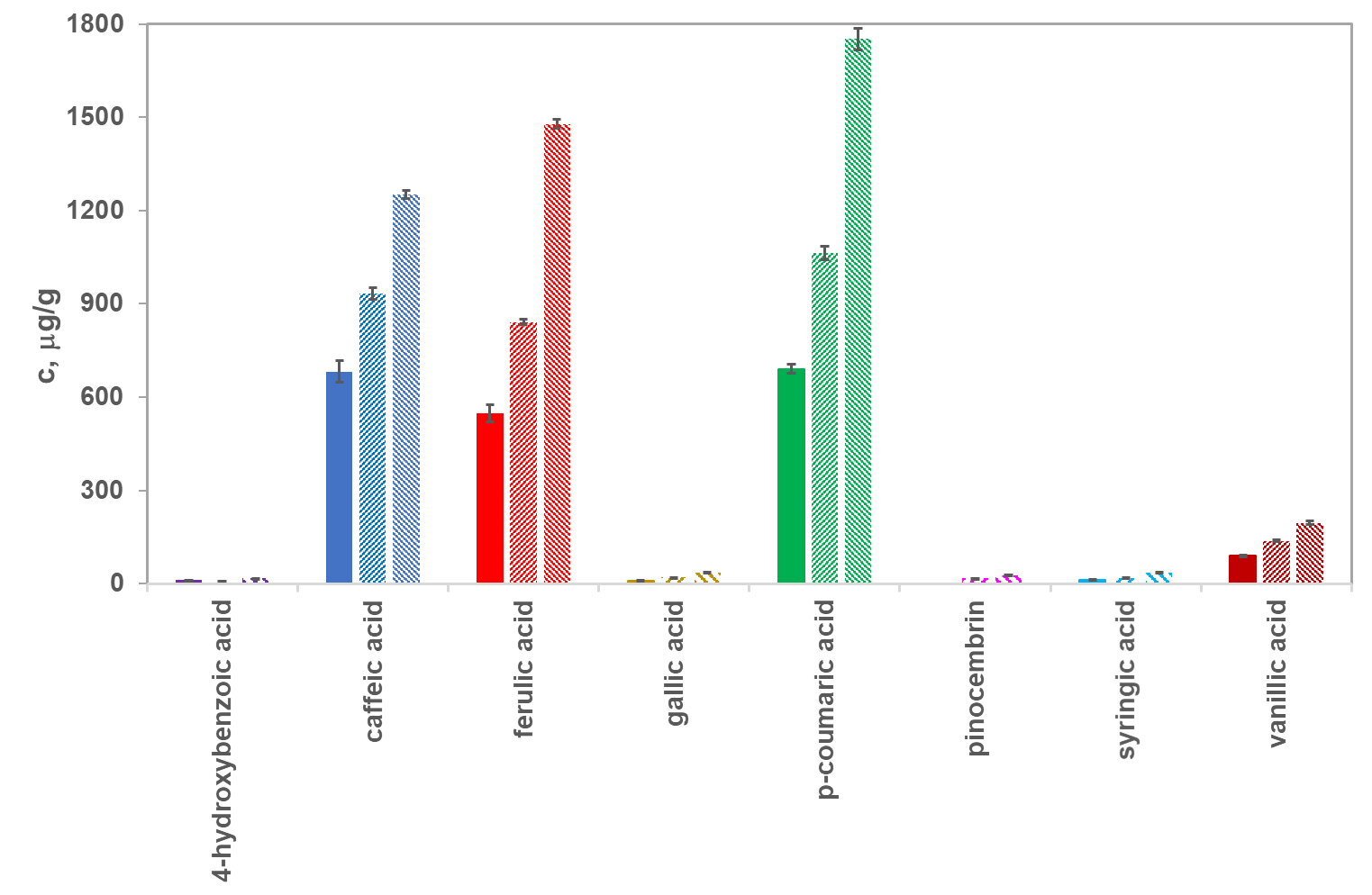


**Figure S3.** Polyphenolics composition pattern at different liquid : solid ratios in water
(A25 – full colour, A45 – diagonal stripes upward, and A65 – diagonal stripes downward).


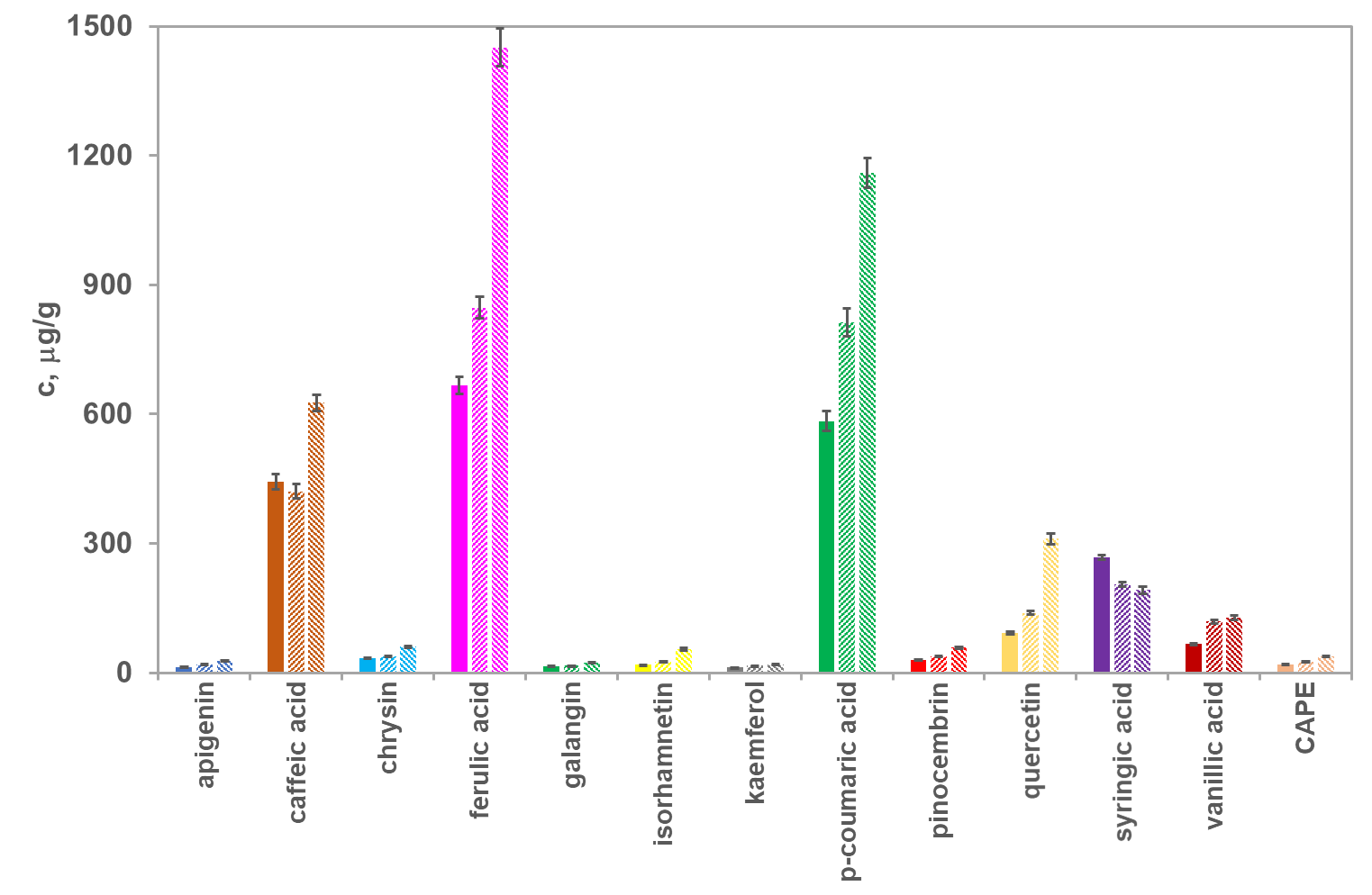


**Figure S4**. Polyphenolics composition pattern at different liquid : solid ratios in 25 % ethanol
(E25 – full colour, E45 – diagonal stripes upward, and E65 – diagonal stripes downward).


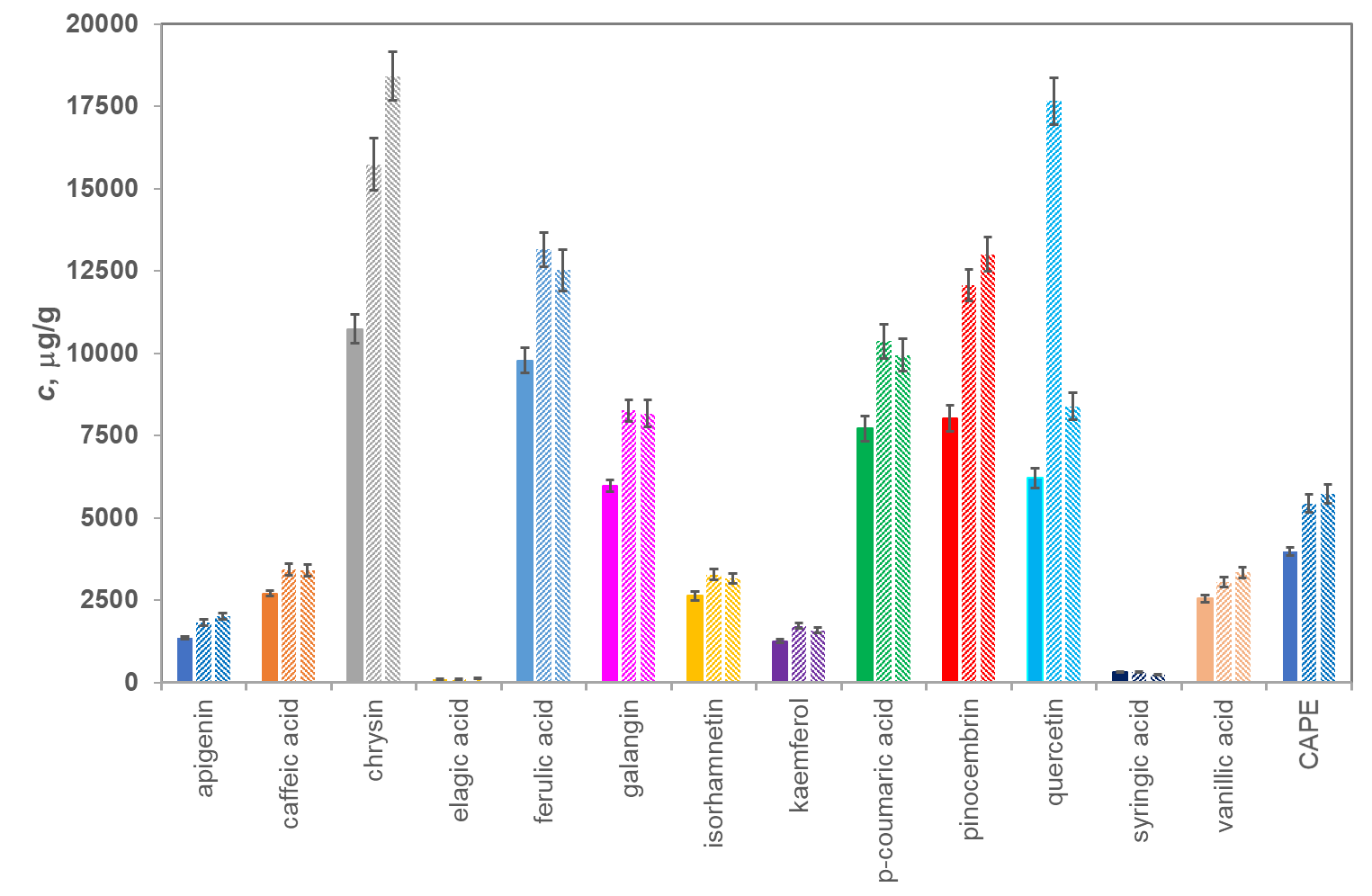


**Figure S5**. Polyphenolics composition pattern at different liquid : solid ratios in 70 % ethanol
(EEE25 – full colour, EEE45 – diagonal stripes upward, and EEE65 – diagonal stripes downward).


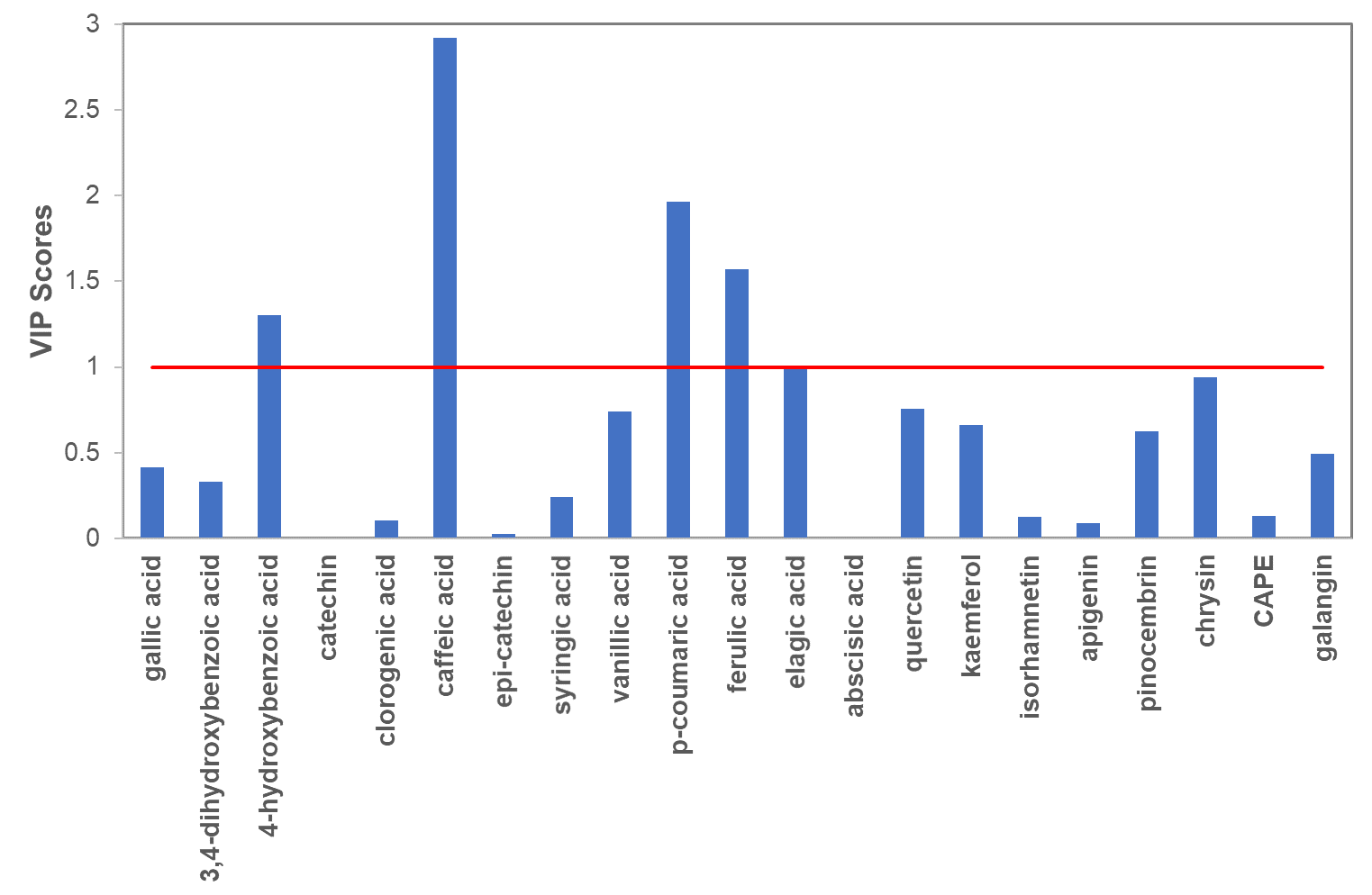


**Figure S6.** Chemical components with important contribution in the PLS model (VIP scores >1) for water extracts.


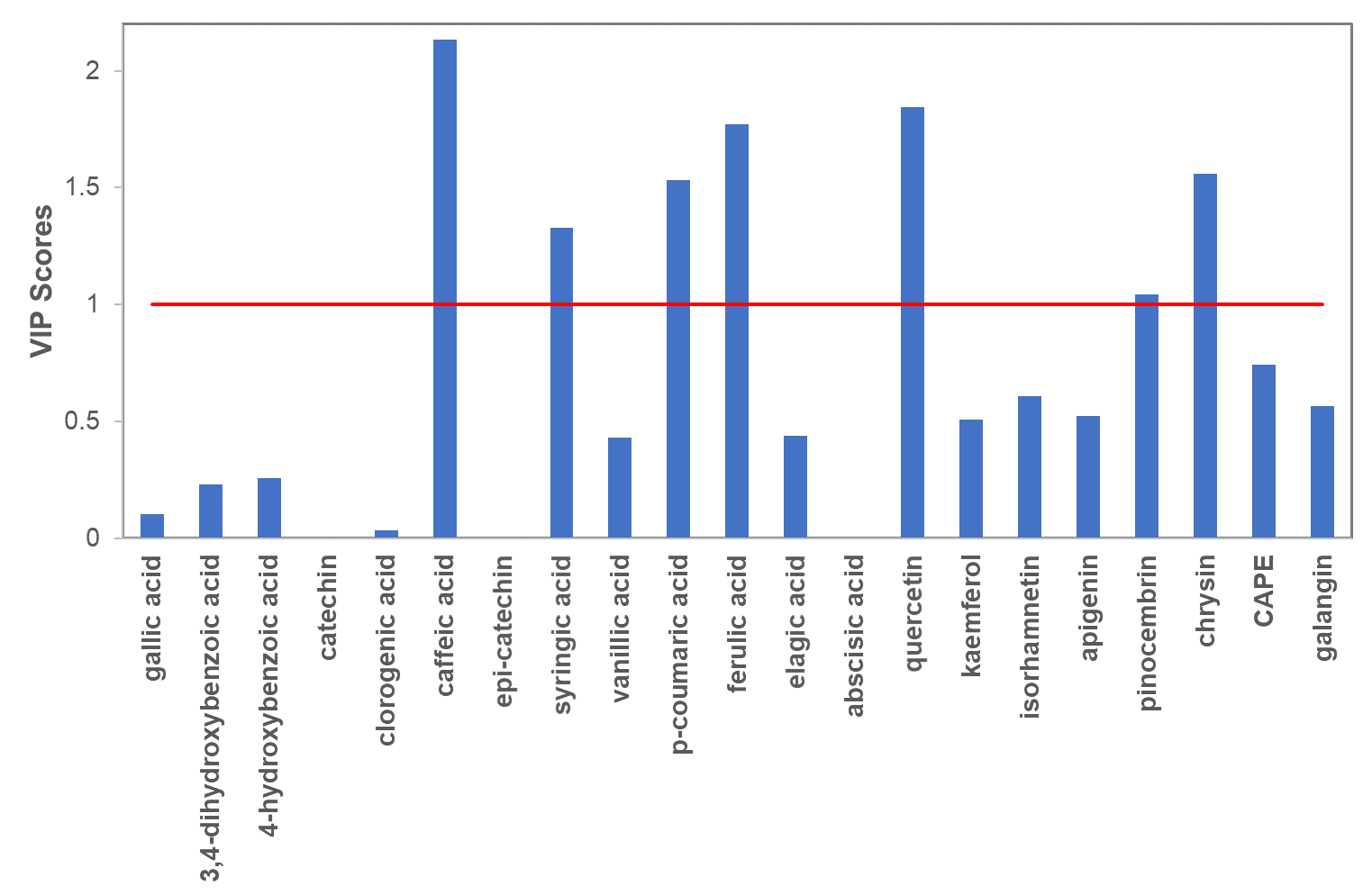


**Figure S7**. Chemical components with important contribution in the PLS model (VIP scores >1) for 25% ethanolic extracts


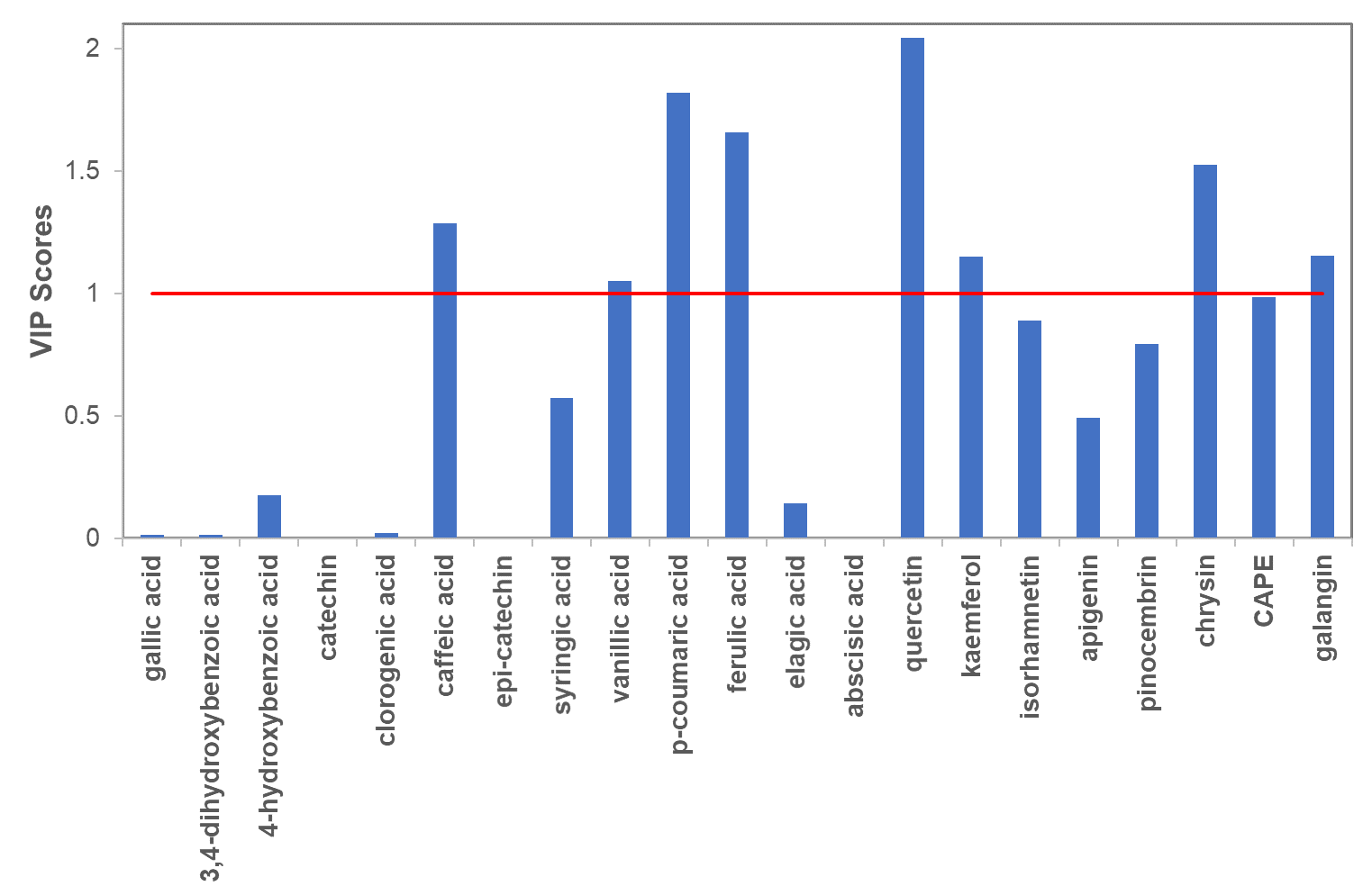


**Figure S8**. Chemical components with important contribution in the PLS model (VIP scores >1) for 50% ethanolic extracts


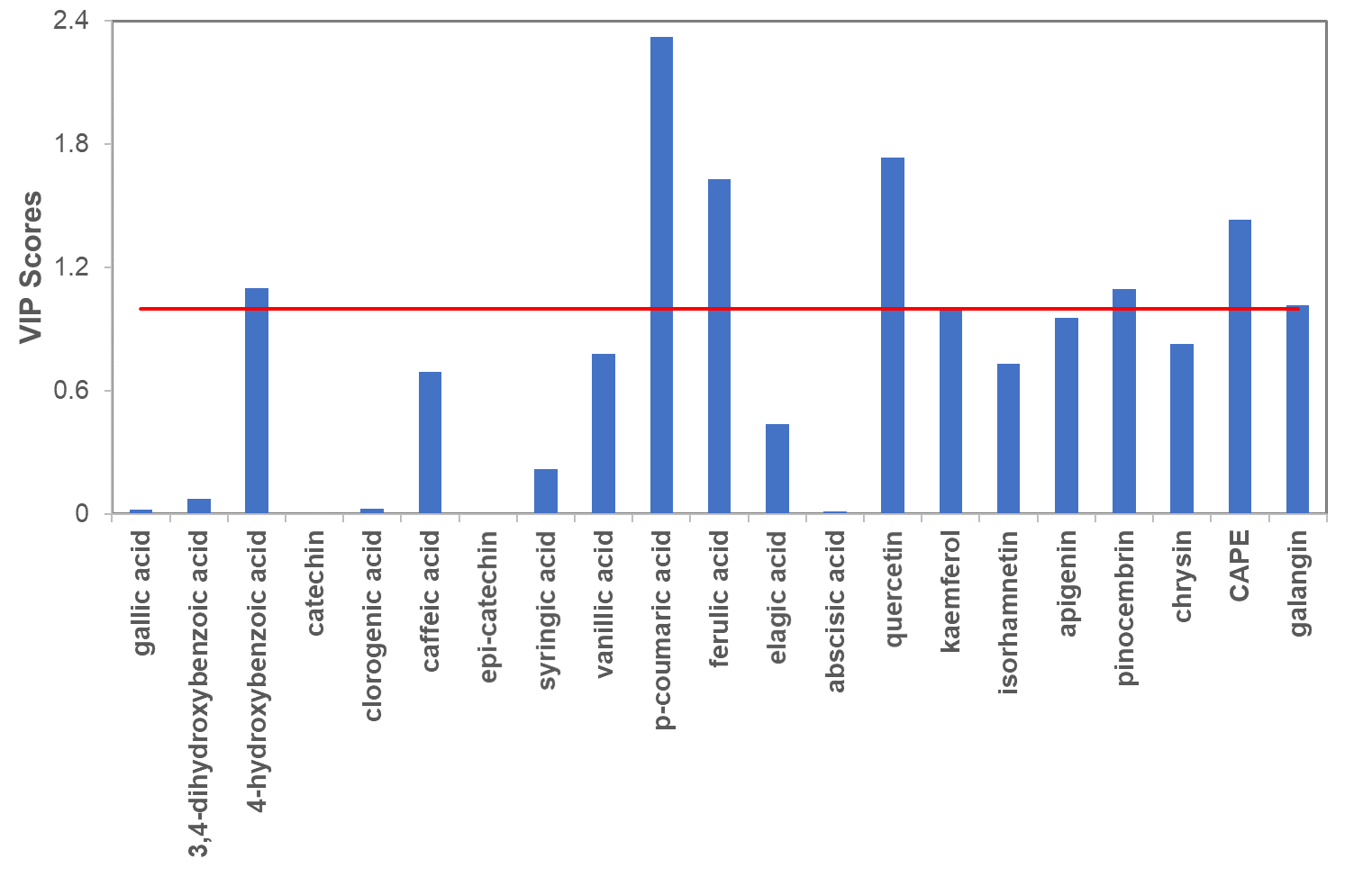


**Figure S9**. Chemical components with important contribution in the PLS model (VIP scores >1) 70% ethanolic extracts**.**

**Saturation model details**

**Table S3**. Saturation model parameters for selected chemical components

| Parameter | Caffeic acid | *p*-Coumaric acid | *t*-Ferulic acid | Chrysin | Galangin | Quercetin | Pino-cembrin | CAPE |
| --- | --- | --- | --- | --- | --- | --- | --- | --- |
| *K_max_*  [μg TEAC/mL] | 24246 | 22800 | 21317 | 13917 | 13866 | 15567 | 14066 | 14857 |
| *K_c_* [μg/mL] | 843 | 2195 | 1459 | 71.7 | 31.8 | 232.9 | 63.8 | 42.2 |
| R^2^ | 0.78 | 0.93 | 0.95 | 0.95 | 0.95 | 0.96 | 0.95 | 0.96 |

The different shapes of the saturation-type model are mainly due to the different extraction capabilities of solvents used for polyphenolic acids and flavones. The very low flavonoids concentration (pinocembrin in Figure **S10 c**) in water and 25% ethanol must be correlated with some increasing antioxidant capacity of these extracts (blue and red points in Figure **S10 c**) which are due to the phenolic acids extracted in such solvents. This is reflected in a steep increase of the curve representing the saturation model for the 50% and 70% ethanol extracts, this capacity slightly varying around 15000-16000 μg/mL. Therefore, the antioxidant potential is attained for rather low concentrations, at a value close to the experimental ones.

If only the data collected from 50% and 70% ethanolic solvents are used in regression analysis, the saturation model indicates a reasonable correlation (Figure **S10** d) and an antioxidant potential of 17300 μg/mL, fact in good agreement with experimental measured values and the antioxidant potential evaluated from the correlation of total phenolic acids and flavonoids concentration with antioxidant capacity (18505 μg/mL). Both Figures **S10 c** and **d** prove that flavonoids, difficult to be extracted in dilute ethanolic solvents, have a high contribution in the rapid increase of the extract’s antioxidant capacity when 50% ethanol solutions are employed.

As for phenolic acids, their concentrations steadily increase in the extracts, in the order

water <25% (w/w) ethanol < 50% (w/w) ethanol <70% (w/w) ethanol

and the correlation model has a less steep increase, consequently showing no asymptotic tendency towards the maximum experimentally measured antioxidant capacity. The higher antioxidant potential identified for these compounds has a mathematical significance as it can be reached only at very high concentrations, which are unlikely to be obtained in propolis extracts unless more efficient extraction techniques are used.

| 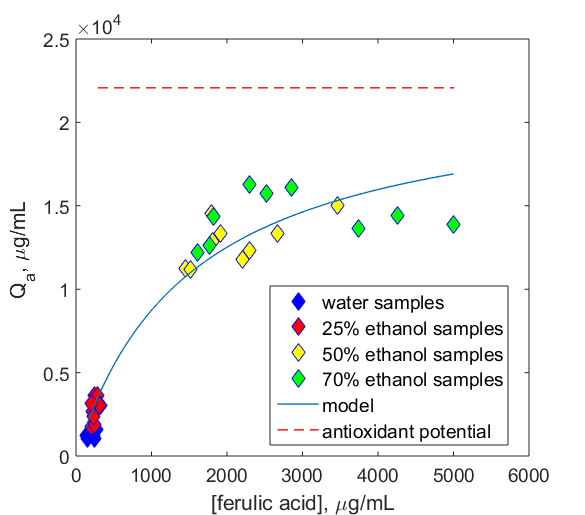(a) | 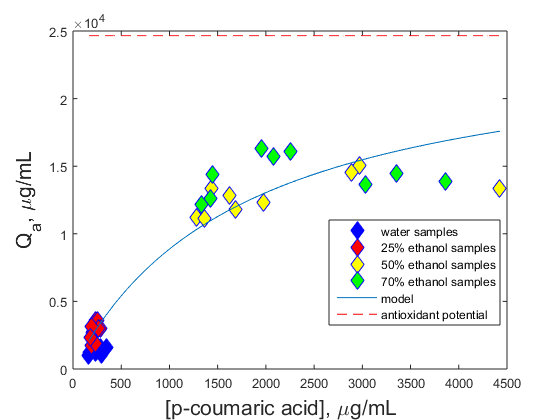(b) |
| --- | --- |
| 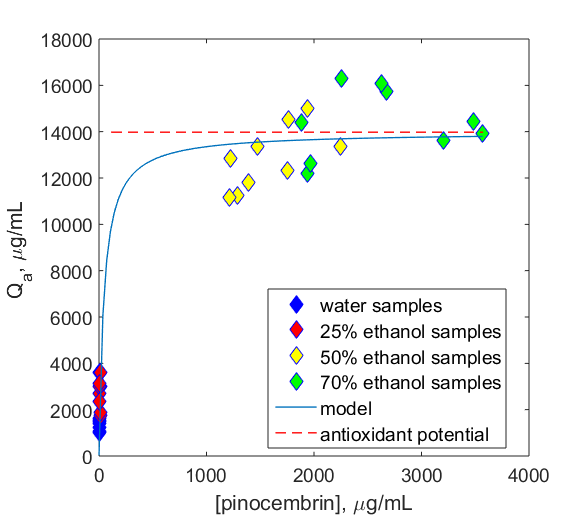  (c) | 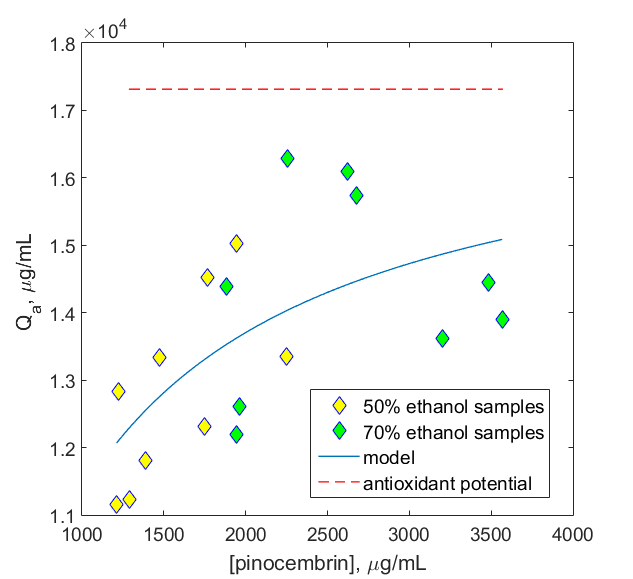  (d) |
| 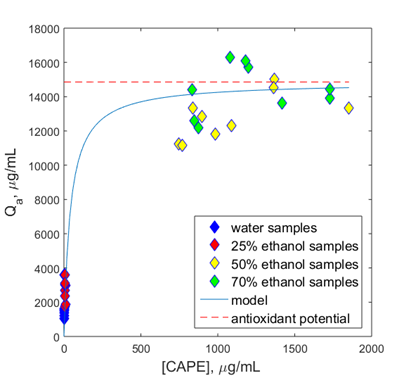  (e) | |

**Figure S10.** Saturation model correlations for some polyphenolic acids and flavonoids.

**Table S4.** Correlation between the component structure and model parameters

| Component | Maximum experimental concentration,  *c*_max_, μg/mL | *K_c_* / *c*_max_ | *K_max_* / *K_c_* |
| --- | --- | --- | --- |
|   *p*-coumaric acid | 4427 | 0.49 | 10.4 |
|   *t*-ferulic acid | 5000 | 0.29 | 14.6 |
|   caffeic acid | 2357 | 0.10 | 29.2 |
| 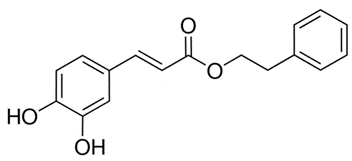  CAPE | 1853 | 0.02 | 352 |
|   quercetin | 3837 | 0.06 | 66.8 |
|   chrysin | 5060 | 0.014 | 194 |
|   pinocembrin | 3567 | 0.017 | 220 |
|  galangin | 2924 | 0.011 | 436 |

| 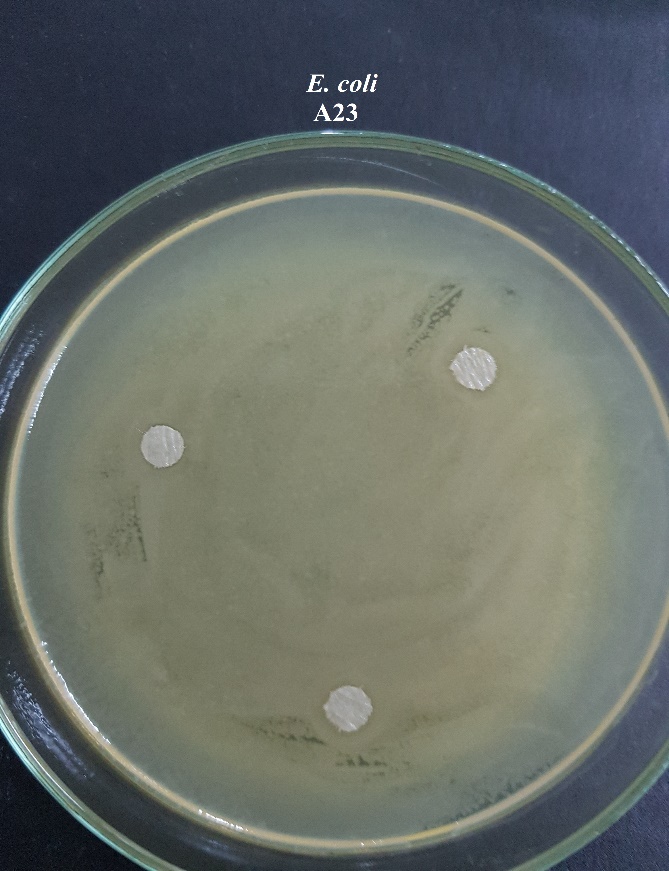 | 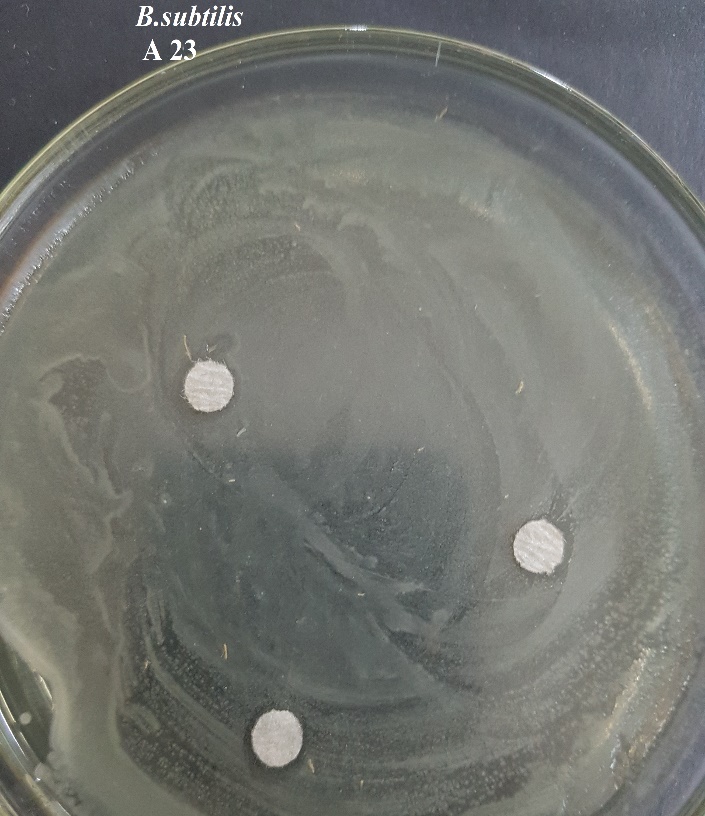 |
| --- | --- |
|  |  |
| 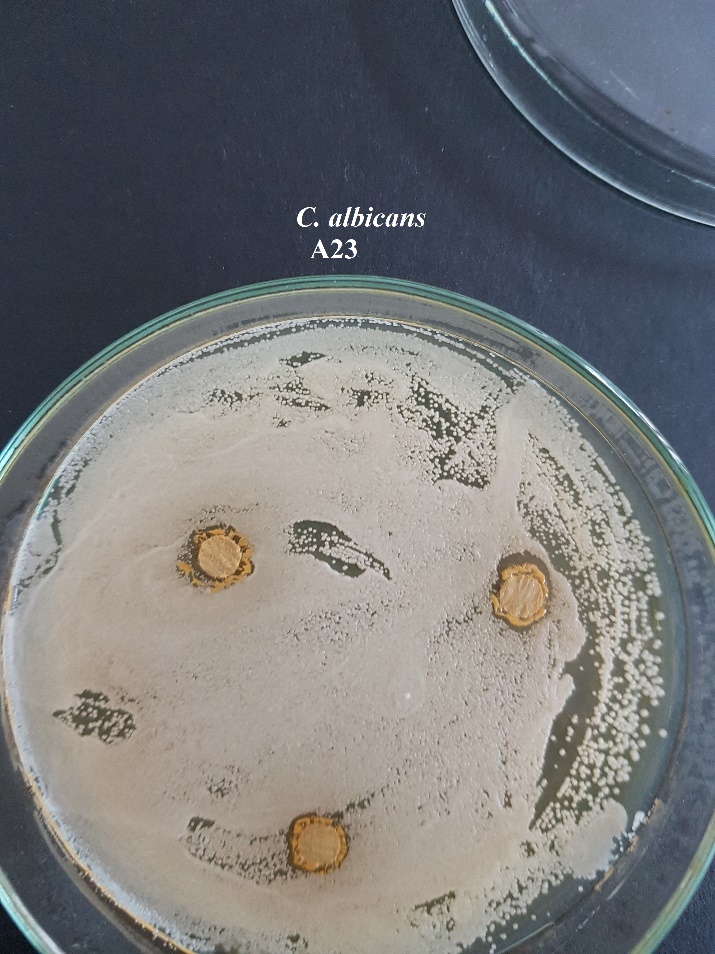 | |
|  | |

**Figure S11.** Antimicrobial activity of aqueous extract of propolis

| 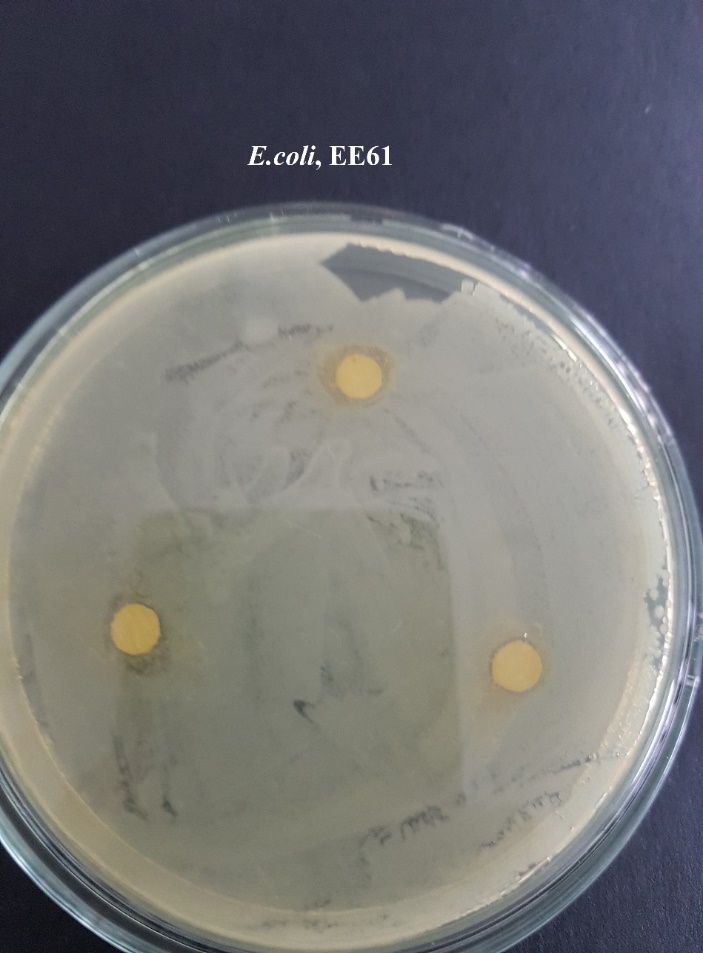 | 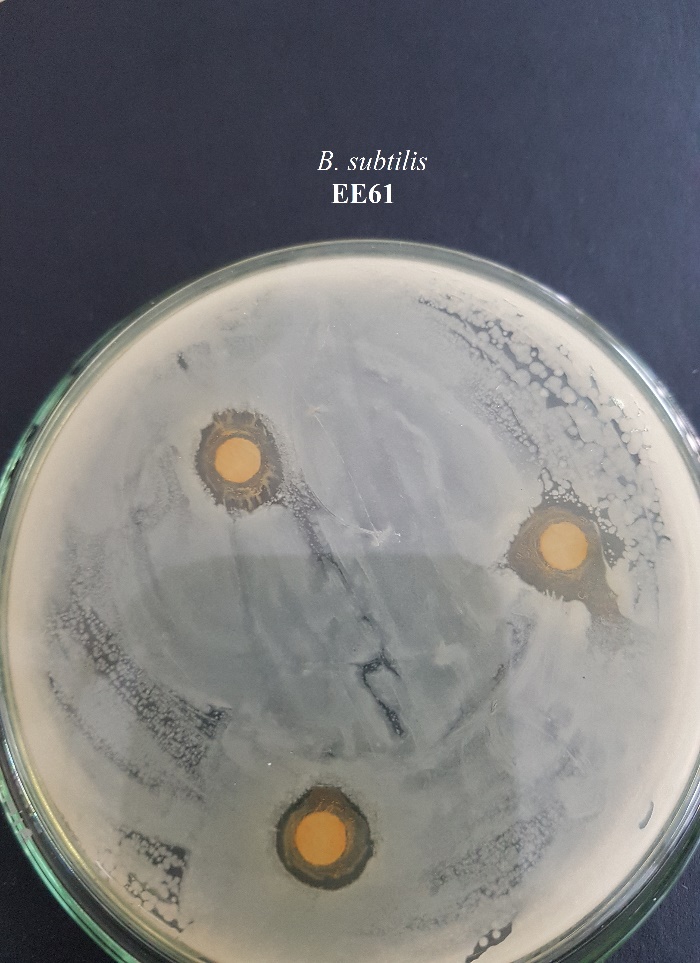 |
| --- | --- |
|  |  |
| 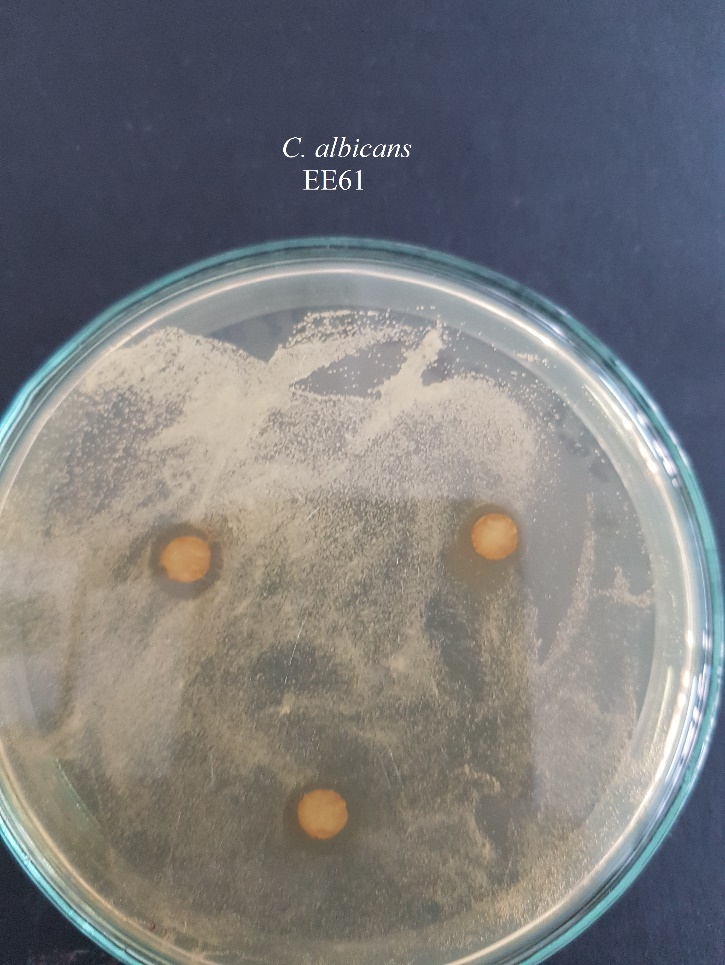 | |
|  | |

**Figure S12.** Antimicrobial activity of 50% ethanolic extract of propolis
